# Fibroblast Common Serum Response Signature-Related Classification Affects the Tumour Microenvironment and Predicts Prognosis in Bladder Cancer

**DOI:** 10.1155/2022/5645944

**Published:** 2022-10-19

**Authors:** Xiangchou Yang, Yangyang Zhou, Linjing Huang, Shang Lin, Haihao Ye, Yujuan Shan

**Affiliations:** ^1^Department of Hematology and Medical Oncology, The Second Affiliated Hospital and Yuying Children's Hospital of Wenzhou Medical University, Wenzhou, 325000 Zhejiang Province, China; ^2^Department of Pathology, The Second Affiliated Hospital and Yuying Children's Hospital of Wenzhou Medical University, China; ^3^Department of Nuclear Medicine, The Second Affiliated Hospital and Yuying Children's Hospital of Wenzhou Medical University, Wenzhou, Zhejiang, 325002, China; ^4^Department of Cardiology, Wenzhou TCM Hospital, Wenzhou 325000, China; ^5^School of Public Health and Management, Wenzhou Medical University, Wenzhou 325035, China

## Abstract

Abnormal oncogenic signatures provide important clues regarding cancer prognosis and treatment. We analysed the variations in 189 oncogenic signature gene sets between normal and tumourous tissues from The Cancer Genome Atlas (TCGA) and found that the “CSR_LATE_UP” signature was the most upregulated oncogenic signature gene set in bladder cancer. Next, we developed a common serum response (CSR) risk score (CRS) model based on fibroblast CSR genes and systematically analysed the correlations of these genes or the CRSs with survival, previously reported molecular subtypes, clinicopathological features, cancer signalling pathways, chemotherapeutic responses, and the tumour microenvironment using TCGA and validation cohorts. The CRS could predict the malignant phenotype, chemotherapeutic efficacy, immune invasion, and disease prognosis. Inflammatory signalling pathways (e.g., inflammatory response, TNFA signalling via NFƘB, IFN*α* response, and IL2-STAT5 signalling) were markedly upregulated in patients with high CRS. Notably, the CSR-related gene *ANLN* was positively correlated with CD8^+^ immune cell infiltration, PD-L1 expression, and sensitivity to PD-L1 inhibitors and could thus provide guidance for clinical immunotherapy. This study highlights the crucial role of the CSR signature in bladder cancer and provides a CRS model for accurate predictions of the disease prognosis and chemotherapy and immunotherapy responses.

## 1. Introduction

Bladder cancer (BLCA), which represents the tenth most common types of cancer worldwide, is the predominant malignancy of the urinary system. The main type of BLCA is uroepithelial carcinoma, which is clinically classified into non-basal-invasive and muscle-invasive subtypes. There are about 573,000 cases of BLCA worldwide, with 212,000 deaths. Furthermore, with the aging of the global population, the morbidity related to this urinary system disease is increasing annually [1]. Currently, surgical resection remains the first treatment of choice for patients with BLCA. Although postoperative platinum-based chemotherapy can improve the prognosis of afflicted patients, resistance to chemotherapeutic drugs is still a difficulty faced in BLCA treatment [2].

Researchers continue to identify oncogenic signature gene sets in different tumour types [3, 4]. Such gene sets indicate the signature of cellular pathways that are often dysregulated in cancer. Classification based on abnormal oncogenic signatures provides important information on the disease prognosis and treatment. For instance, Zhang et al. introduced a novel metabolic classification method based on genes related to glycolysis and the cholesterol synthesis pathways in patients with BLCA, which might contribute to precision therapeutic strategies targeting metabolic weaknesses [5]. Thus, the identification of the important abnormal signalling pathways in BLCA could lead to similar novel strategies for combating the disease. To systematically investigate the core gene sets involved in BLCA development and progression, we downloaded all oncogenic signature gene sets from the Molecular Signatures Database (MSigDB) and analysed their variations between normal and tumourous tissues, whereupon “CSR_LATE_UP.V1_UP” (a common serum response (CSR) signature) was found to be the most upregulated in the disease samples. Containing 166 upregulated CSR genes, this signature was first discovered in the late serum response of foreskin fibroblasts, which seems to represent the pleiotropic roles of fibroblasts in wound healing. Chang et al. connected this CSR signature to cancer progression and determined the prognostic performance of the CSR signature in breast cancer and epithelial tumours [6]. However, there is a dearth of publications on the role of CSR signatures in BLCA development and progression. Thus, this study was carried out to identify the CSR genes which have value in predicting the progression of BLCA and the prognosis of afflicted patients.

Recent developments in tumour immunotherapy have brought new hope to patients with BLCA. Although the use of immune checkpoint blockades (ICBs) has significantly improved the outcome of cancer patients, only a minority of patients with BLCA respond effectively to this type of therapy [7]. The immunotherapy response depends on the number of tumour-infiltrating lymphocytes (TILs) [8]. Moreover, the incidence of adverse reactions to anti-programmed cell death ligand 1 (PD-L1) therapy is approximately 16% [9]. To reduce both the financial burden of cancer patients and the side effects of drugs, individualised therapy is needed to achieve more effective and economical treatment. Therefore, the discovery of immune detection targets provides a basis for the choice of ICB therapy in patients with BLCA. Although tests of the tumour mutational burden, microsatellite instability, and molecular subtypes are helpful in predicting the clinical response to ICBs, they are time-consuming and expensive. Thus, it is of great importance to find new, economical, simple, and effective immune molecules that can act as prognostic indicators.

In this study, we analysed the variations in oncogenic signature gene sets between 19 normal and 405 tumourous tissues from The Cancer Genome Atlas (TCGA-BLCA) and found that the CSR signature was the most upregulated oncogenic signature gene set in BLCA. On the basis of these CSR genes, we built a CSR risk score (CRS) model to predict the outcome of patients with BLCA. From the results of the correlation analyses between the CRS classifications and clinicopathological features or immunity characteristics of the tumour microenvironment, we propose that the model can predict tumour progression and thus could be used to guide the choices of chemotherapy and PD-L1-targeted immunotherapy treatment for patients with BLCA.

## 2. Methods

### 2.1. Data Source

**Figure 1:**
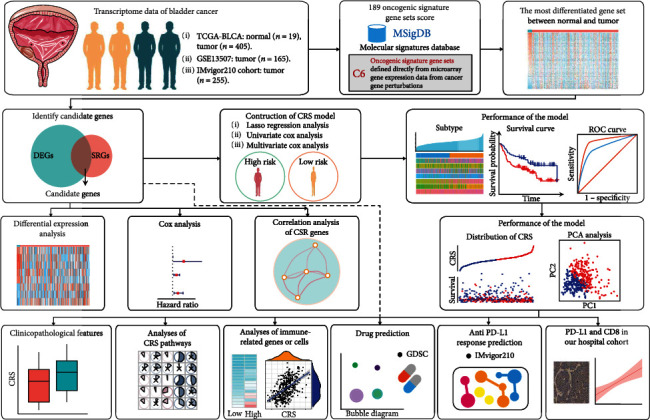
The overall flowchart of this study. DEGs: differentially expressed genes; SRGs: survival-related genes; CRS: fibroblast common serum response risk score.

The overall outline of this study is shown in Figure 1. TCGA-BLCA data were downloaded from https://portal.gdc.cancer.gov/, which contains 19 normal tissues and 405 BLCA tumourous tissues. The dataset of the GSE13507 cohort was downloaded from https://www.ncbi.nlm.nih.gov/geo/query/acc.cgi?acc=GSE13507. Gene expression data and clinical information on the IMvigor210 cohort (a BLCA cohort containing responses to anti-PD-L1 immunotherapy) were obtained from http://research-pub.Gene.com/imvigor210corebiologies/ [10].

### 2.2. Identification of the Most Differential Gene Set

A list of 189 oncogenic signature gene sets was acquired from MSigDB (http://www.gsea-msigdb.org/gsea/msigdb/index.jsp). Gene set variation analysis (GSVA), which is a method used to assess variations in pathway activity [11], was used to evaluate the activities of the 189 oncogenic signature gene sets in the normal and tumourous BLCA tissues. Then, on the basis of the 189 gene set scores, variation analysis between the normal and tumourous tissues was performed using the Limma package. The “CSR_LATE_UP.V1_UP” gene set, which was also downloaded from MSigDB, contains 166 CSR genes that are upregulated in the late serum response of fibroblasts.

### 2.3. Construction of the CRS Model

Before developing the CRS model, we conducted variation analysis of the CSR genes between the normal and tumourous tissues and Cox regression analysis of the differentially expressed genes in relation to BLCA prognosis. Then, the CSR genes that were differentially expressed and had prognostic value were considered as candidates for use in the model construction. By imposing a regression penalty on all variables, the CRS model was constructed to yield a coefficient of zero for the relatively unimportant variables (which were excluded from the model), using the least absolute shrinkage and selector operation (LASSO) algorithm:

(1) $$\begin{array}{c} \text{CRS}=\sum \beta i^{*}\chi i, \end{array}$$

where *βi* is the coefficient of the log_2_-transformed gene expression level for gene *i*. Thereafter, the patients in TCGA and validation cohorts were classified into high-CRS (CRS ≥ median value) and low-CRS (CRS < median value) groups according to the CRS.

### 2.4. Enrichment Analysis of Related Signalling Pathways

The following are the signalling pathways included in this analysis: cell cycle, negative apoptosis, positive apoptosis, fibroblast growth factor receptor (FGFR) activated, Hippo activated, Notch activated, phosphoinositide 3-kinase (PI3K) activated, transforming growth factor-beta (TGF-*β*) activated, Wnt activated, RAS activated, Hippo repressed, Notch repressed, PI3K repressed, Wnt repressed, RAS repressed, basal differentiation, epithelial-mesenchymal transition (EMT) differentiation, immune differentiation, interferon response, keratinisation, luminal differentiation, myofibroblasts, neuroendocrine differentiation, and Ta pathway. These are all important cancer signalling pathways or characteristic pathways of BLCA [12]. The variation in pathway activity was calculated using the GSVA method. Additionally, the biological processes associated with high and low CRSs in the training and validation cohorts were analysed using gene set enrichment analysis (GSEA). The hallmark gene set of “h.all.v7.5.1. entrez.gmt” was acquired from MSigDB.

### 2.5. Evaluation of Immune Cell Infiltration and Function

We also used the GSVA method to evaluate the abundance of immune cell infiltration and immune function. We identified immune subtypes on the basis of the immune landscape of cancer previously reported by Thorsson et al. [13]. The immune and stromal scores were evaluated by the ESTIMATE algorithm, which allows one to infer the proportion of stromal and immune cells in the tumour samples [14].

### 2.6. Prediction of Drug Responses

We downloaded drug response and cell line expression data from the Genomics of Drug Sensitivity in Cancer (GDSC, https://www.cancerrxgene.org/downloads/) [15]. Analyses of the correlation between cell-line-specific gene expression and chemotherapy sensitivity were conducted. Drugs with a *P* value of less than 0.05 are shown in the bubble diagram. pRRophetic is an R package that aids in the prediction of clinical drug responses from tumour gene expression levels [16]. The difference in drug sensitivity between the high- and low-CRS groups was predicted using the pRRophetic package.

### 2.7. Immunohistochemical Analysis

In total, 21 BLCA tissue samples were acquired from patients who had undergone curative surgery at the Second Affiliated Hospital of Wenzhou Medical University. Ethical approval was obtained from the Second Affiliated Hospital of Wenzhou Medical University Research Ethics Committee. Antibodies against anillin (ANLN) (DF13590, 1 : 200; Affinity Biosciences), CD8 (PB9249, 1 : 200; BOSTER), and PD-L1 (66248-1-lg, 1 : 5000; Proteintech) were used for the immunohistochemical (IHC) staining of these proteins in the tissue samples, which was performed according to previously published methods [17]. For the IHC analysis, the *H*-score was applied to assess the expression levels of PD-L1 and ANLN [18]. The number of CD8+ cells was counted using the ImageJ software.

### 2.8. Cancer Dependency Map

The gene effects of 29 BLCA cell lines were obtained from the Cancer Dependency Map (DepMap) database (https://depmap.org/portal/download/custom/). The gene effect score reflects the dependency of a cell on a gene, where a lower score indicates that a cell is more likely to rely on the gene. A gene effect score of 0 indicates a nonessential gene, whereas a score of –1 is the median of all common essential genes.

### 2.9. Statistical Analyses

Pearson's correlation coefficient was used to measure the association between variables. The significance of the difference between two groups of variables was estimated using Student's *t*-test. The *χ*^2^ was used to analyse the correlation between the risk stratification of CSR genes and types of BLCA. The Kaplan–Meier method and log-rank test were applied to assess the statistical significance of the prognostic classification variables of survival curves. The “timeROC” R package was used to plot receiver operating characteristic curves. All statistical analyses were performed using R version 4.0.2. A *P* value of less than 0.05 was considered statistically significant.

## 3. Results

### 3.1. Identification of the Most Differential Oncogenic Signature Gene Sets and Related Genes in Bladder Cancer

**Figure 2:**
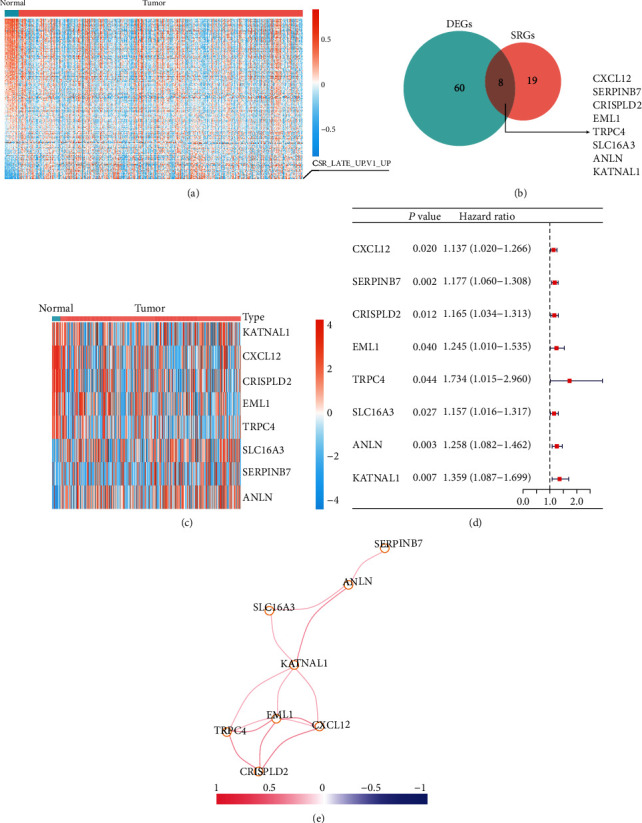
Identification of most differential oncogenic signature gene sets and related genes. (a) Heat map of 189 oncogenic signature gene sets between normal and tumourous tissues of bladder cancer. (b) Eight fibroblast common serum response- (CSR-) related genes differentially expressed in bladder cancer showed prognostic value. (c) Heat map of mRNA levels of the identified eight CSR genes between normal and tumourous tissues in bladder cancer. (d) Forest map shows the hazard ratio of the identified eight CSR genes in TCGA-BLCA cohort. (e) Correlation analysis among the identified eight CSR genes. DEGs: differentially expressed genes; SRGs: survival-related genes.

To investigate the crucial oncogenic signature gene sets in BLCA, we performed variation analysis between the normal and tumourous tissues. The results showed that “CSR_LATE_UP.V1_UP” was the most upregulated oncogenic signature gene set in BLCA (Figure 2(a) and Supplementary Table 1). Next, we analysed the variations in CSR genes between normal and tumourous tissues and conducted a Cox regression analysis of the differentially expressed genes in relation to BLCA prognosis (Supplementary Tables 2 and 3). Only eight genes were differentially expressed in BLCA and associated with prognosis, namely, the genes encoding C-X-C motif chemokine ligand 12 (*CXCL12*), Serpin family B member 17 (*SERPINB7*), cysteine-rich secretory protein LCCL domain-containing 2 (*CRISPLD2*), EMAP-like 1 (*EML1*), transient receptor potential cation channel subfamily C member 4 (*TRPC4*), solute carrier family 16 member 3 (*SLC16A3*), *ANLN*, and katanin catalytic subunit A1-like 1 (*KATNAL1*) (Figure 2(b)). Among these genes, *KATNAL1*, *CXCL12*, *CRISPLD2*, *EML1*, and *TRPC4* were downregulated in BLCA, whereas *SLC16A3*, *SERPINB7*, and *ANLN* were upregulated (Figure 2(c)). Cox regression analysis revealed that all eight CSR genes were risk factors for poor prognosis in BLCA (Figure 2(d)). Generally, positive correlations were observed between the expression of the eight genes (Figure 2(e)).

### 3.2. Construction and Validation of the CRS Model

**Figure 3:**
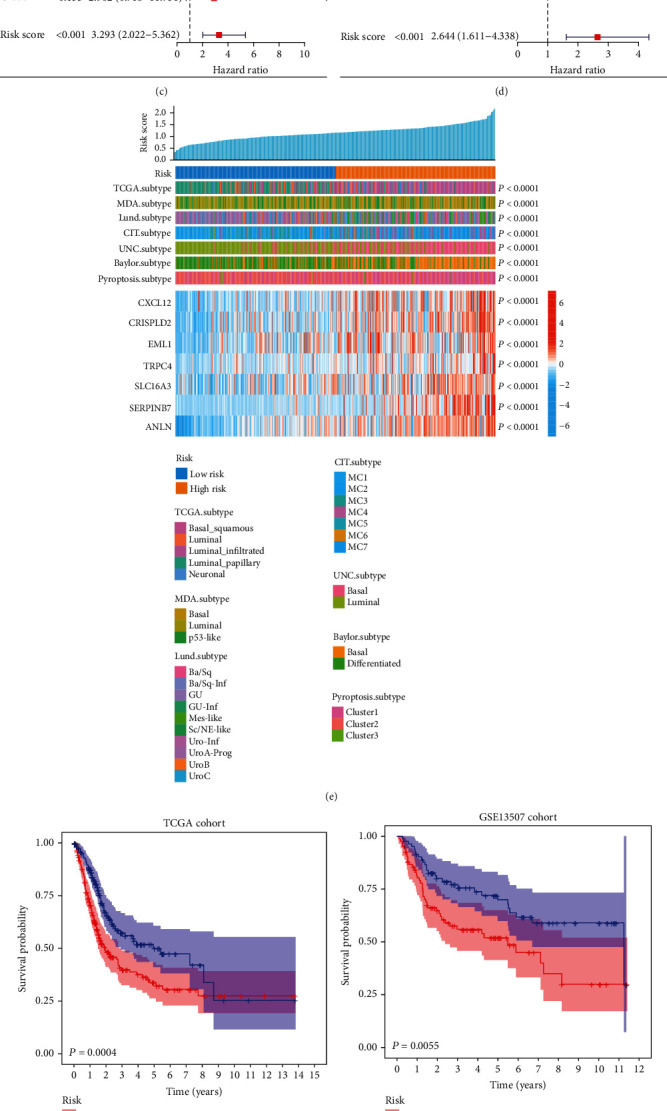
Construction of CRS model using the identified eight CSR genes. (a) Cross-validation for turning parameter selection via minimum criteria in the LASSO regression model. The penalty value is determined according to the lowest point of the curve (the upper coordinate corresponding to the lowest point of the curve). (b) Coefficient profiles of eight genes in the TCGA-BLCA cohort. The variable that intersects the penalty value is the variable eventually included in the model, and the vertical coordinate corresponding to the variable is the regression coefficient of the variable. (c) Univariate and (d) multivariate Cox analysis of clinical features and the CRS. (e) Correlation between the CRS stratification of CSR genes and types of bladder cancer and the mRNA levels of CSR genes. Ability to prognosticate the survival for patients in the (f) TCGA-BLCA and (g) validation cohorts of the CRS model. Predictive accuracy of the model for survival in the (h) TCGA-BLCA and (i) validation cohorts. CRS: fibroblast common serum response risk score.

As described in Section 3.1, eight CSR genes were identified as candidates for building a CRS model using the LASSO algorithm. Finally, a seven-CSR gene-based risk score was developed: CRS = 0.069^∗^CXCL12 + 0.086^∗^SERPINB7 + 0.017^∗^CRISPLD2 + 0.107^∗^EML1 + 0.214^∗^TRPC4 + 0.066^∗^SLC16A3 + 0.145^∗^ANLN (Figures 3(a) and 3(b)). The univariate and multivariate Cox proportional hazard models indicated that the CRS was an independent risk factor for BLCA (univariate Cox: hazard ratio (HR), 3.293; 95% confidence interval (CI), 2.022–5.362; *P* < 0.001; multivariate Cox: HR, 2.644; 95% CI, 1.611–4.338; *P* < 0.001; Figures 3(c) and 3(d)). Next, the patients in TCGA and validation cohorts were classified into high-CRS (CRS ≥ median value) and low-CRS (CRS < median value) groups (Supplementary Tables 4 and 5). Then, we compared the risk stratifications of several previously reported molecular subtypes of BLCA. Compared with TCGA subtype, the basal squamous subtype was the primary type in the high-CRS group, whereas the luminal papillary subtype was the primary type in the low-CRS group. Compared with the MDA subtype, the basal subtype was predominant in the high-CRS group, and the luminal subtype was predominant in the low-CRS group. Compared with the Lund subtype, the Ba/Sq and Ba/Sq-Inf subtypes were dominant in the high-CRS group, and the UroA-prog subtype was dominant in the low-CRS subgroup. Compared with the CIT subtype, the MC4 and MC7 subtypes were predominant in the high-CRS subgroup, whereas the MC1 subtype was the major type in the low-CRS subgroup. Compared with the Baylor subtype, the basal subtype was of the major type in the high-CRS subgroup, and the differentiated subtype was the main type in the low-CRS subgroup (Figure 3(e)). As shown in Figures 3(f) and 3(g), patients with BLCA in the high-CRS group had an unfavourable outcome compared with those in the low-CRS group in the training (*P* = 0.0004) and validation (*P* = 0.0055) cohorts. The areas under the receiver operating characteristic curves (AUCs) of CRSs were 0.655, 0.622, and 0.634 in 1, 2, and 3 years, respectively, for the training cohort (Figure 3(h)). Similarly, the AUCs of CRSs were 0.676, 0.658, and 0.657 in 1, 2, and 3 years, respectively, for the validation cohort (Figure 3(i)).

### 3.3. Correlation between CRS Stratification and Clinicopathological Features

**Figure 4:**
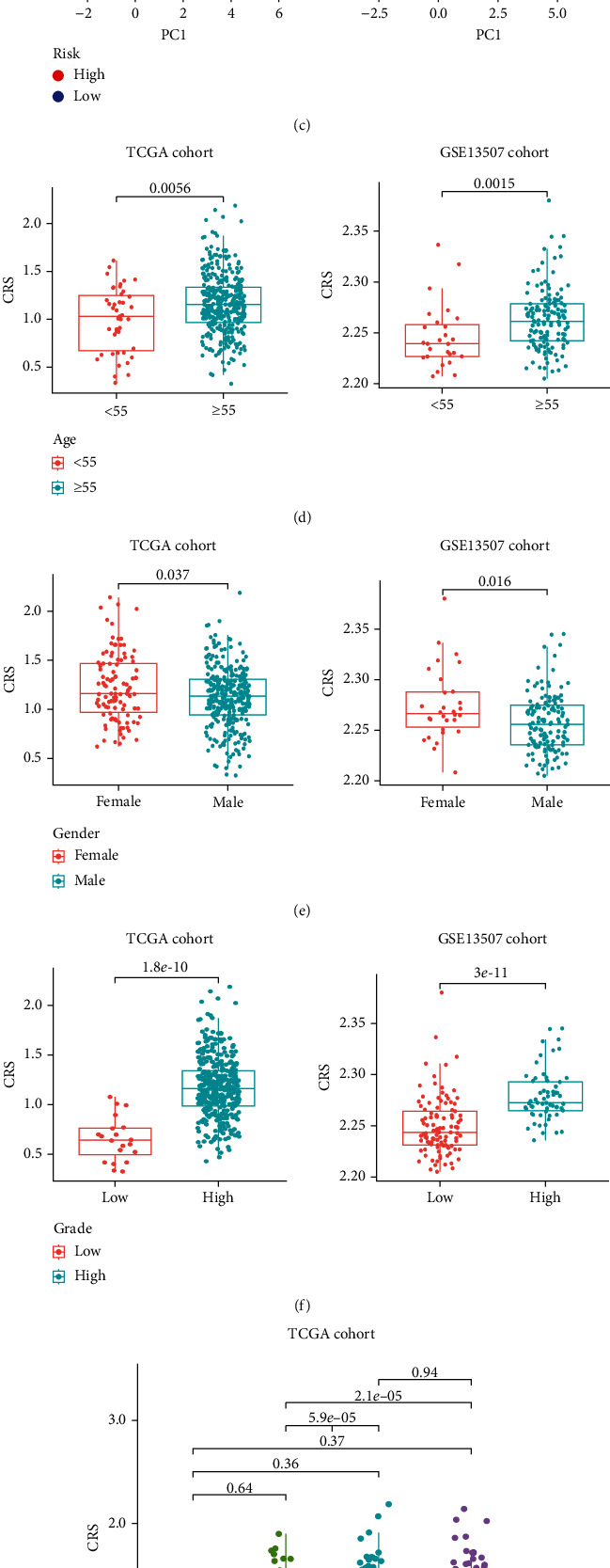
Correlation between the CRS stratification and clinicopathological features. Distribution of the CRS and survival in the (a) TCGA-BLCA and (b) validation cohorts. (c) Principal component analysis shows stratification of the CRS model in the both cohorts. Box plots show the difference in the CRS of (d) age, (e) gender, (f) grade, and (g) TNM staging. CRS: fibroblast common serum response risk score.

The distribution of CRSs and survival periods in TCGA (Figure 4(a)) and validation cohorts (Figure 4(b)) indicated that the patients in the high-CRS subgroup had a shorter survival time. Principal component analysis indicated that the CRS distinguished the two subgroups well in both cohorts (Figure 4(c)). Next, we performed a correlation analysis between CRS stratification and clinicopathological features. Patients aged ≥55 years, female patients, and patients with high-grade tumours had higher CRSs (Figures 4(d)–4(f)). Moreover, patients with a high TNM stage had a higher CRS than those with a low TNM stage in both cohorts (Figure 4(g)).

### 3.4. Correlation between CRS Stratification and Enrichment of Related Signalling Pathways

**Figure 5:**
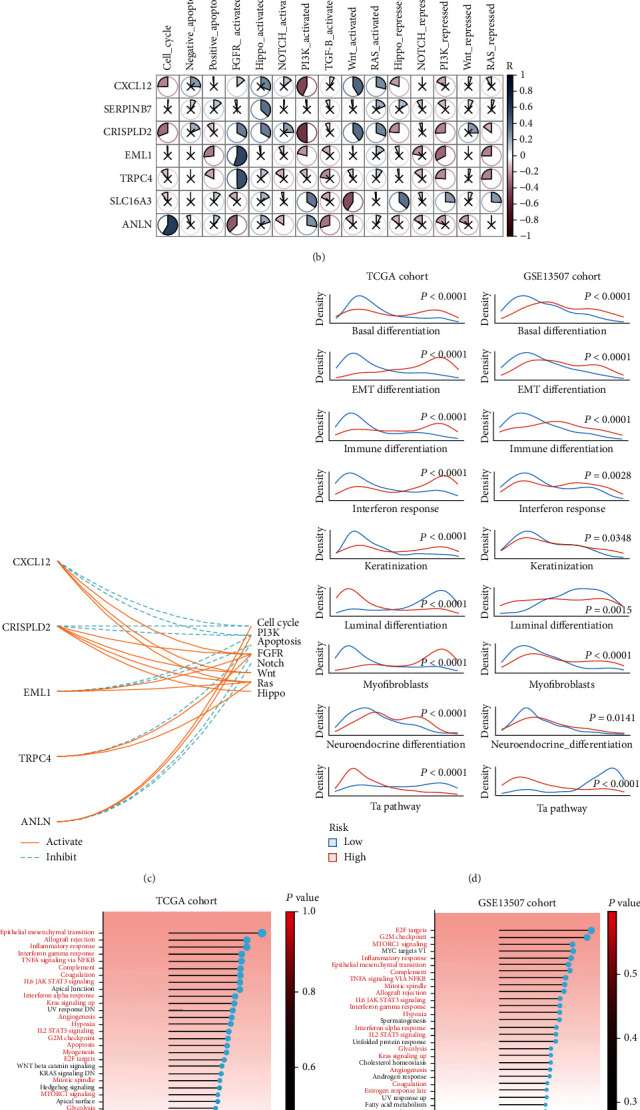
Analyses of CRS-related signalling pathways. Correlation between expressions of seven CSR genes involved in the CRS and important cancer signalling pathways in the (a) TCGA-BLCA and (b) validation cohorts. A cross in the pie chart shows *P* value > 0.05. (c) Wire map outlines the correlation between CSR genes and important cancer signalling pathways with statistical significance in the both cohorts. Solid lines represent activation of signalling pathways and dotted lines show inhibition. (d) Density map shows the difference of characteristic pathway scores between the high- and low-CRS subgroups in BLCA. GSVA enrichment analyses of high- and low-CRS-associated biological processes in the (e) TCGA-BLCA and (f) validation cohorts. The red font indicates common enriched processes in the cohorts. CRS: fibroblast common serum response risk score.

We performed correlation analyses between the CSR genes and important cancer signalling pathways in TCGA (Figure 5(a)) and validation (Figure 5(b)) cohorts (Supplementary Table 6). According to the results, *CXCL12* expression was negatively associated with the cell cycle and PI3K signalling pathways and may activate the FGFR, Wnt, and Ras signalling pathways (Figure 5(c)). Generally, the FGFR, Wnt, Ras, and Hippo signalling pathways were positively related to the CSR genes, whereas apoptosis was inhibited by the upregulation of *EML1* and *TRPC4* expression (Figure 5(c)). When the differences in BLCA characteristic pathway scores between the high- and low-CRS subgroups were investigated, we observed that the basal differentiation, EMT differentiation, immune differentiation, interferon response, keratinisation, myofibroblast, and neuroendocrine differentiation pathways were highly enriched in the high-CRS subgroup, whereas the luminal differentiation and Ta pathways were high in the low-CRS group in the two cohorts (Figure 5(d) and Supplementary Table 7). GSEA software analysis of the biological processes associated with high and low CRSs in TCGA (Figure 5(e)) and validation (Figure 5(f)) cohorts revealed 21 common signalling pathways were upregulated in the high-CRS subgroup in both cohorts. Among them, inflammatory signalling pathways (e.g., the inflammatory response, IFN*γ* response, tumour necrosis factor-alpha signalling via nuclear factor kappa-B, interleukin (IL) 6–Janus kinase (JAK)–signal transducer and activator of transcription (STAT) 3 signalling, IFN*α* response, and IL2–STAT5 signalling) were significantly upregulated in the high-CRS subgroup (Figures 5(e) and 5(f)).

### 3.5. Correlation between the CRS Stratification and Immune-Related Genes or Cells

**Figure 6:**
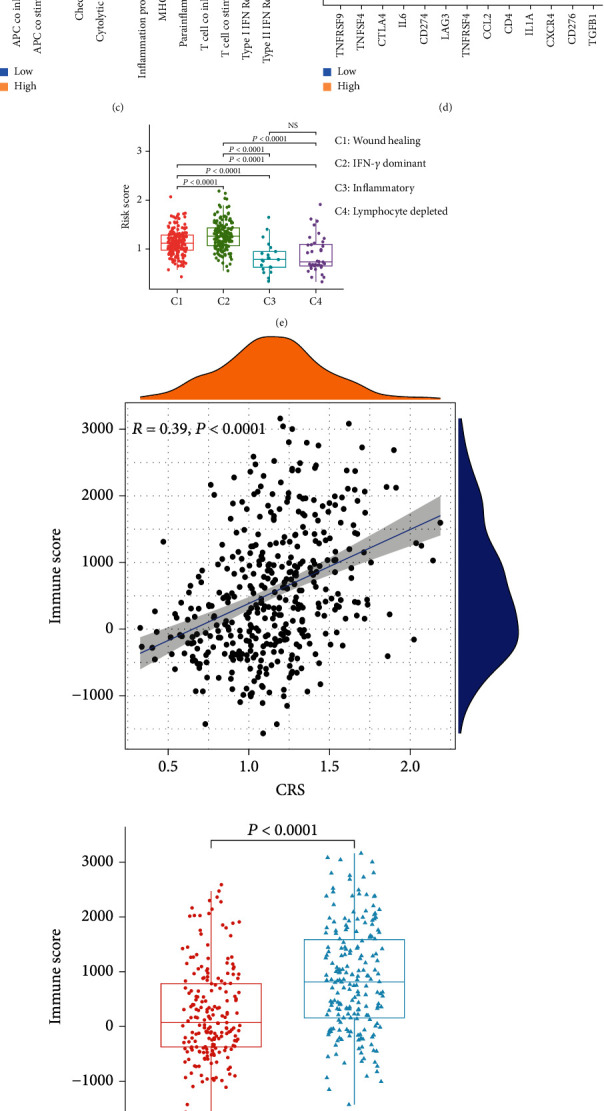
The CRS stratification and immune-related genes or cells. (a) Heat map shows difference of chemokines, interleukins, interferons, other cytokines, and their receptors. Violin plots of the (b) abundance of immune cells and (c) functions of chemokines, interleukins, interferons, other cytokines, and their receptors. (d) mRNA levels of 13 immune checkpoint genes. ns: not significant. (e) Abundance of the risk score in different immune subtypes. Correlation analyses between the CRS and (f) immune score or (g) stromal score. CRS: fibroblast common serum response risk score.

We investigated the connection between the CRS classification and immune cell activity in the tumour microenvironment. According to the heat map generated, several chemokines and receptors were elevated in the high-CRS subgroup (e.g., chemokine (C–C motif) ligand (CCL) 5, C-X-C chemokine receptor type (CXCR) 4, CXCL9, CXCL14, CCL18, CCL21, CXCL11, and CXCL12), which may promote the infiltration of immune cells, such as CD8^+^T cells and dendritic cells (DCs) (Figure 6(a)). Moreover, the expression levels of interleukins, interferons, and other cytokines which may modify immune function were increased in the high-CRS subgroup (Figure 6(a)). Thus, we compared the immune cells, immune function, and immune checkpoint genes between the high- and low-CRS subgroups. Several immune cells (aDCs, B cells, CD8+ T cells, DCs, and T helper cells) were observably enriched in the high-CRS subgroup (Figure 6(b)). Through the analysis of immune functions, we found that antigen-presenting cell (APC) costimulation, immune checkpoints, and MHC class I were also significantly elevated in the high-CRS subgroup (Figure 6(c)). Thus, for the two CRS subgroups, we further investigated their variations in 13 potentially targetable immune checkpoint genes whose drug inhibitors are being used in clinical trials or have been approved for use in some cancer types. Except for that of *IL1A*, the expression levels of the other 12 genes were obviously upregulated and were observably elevated in the high-CRS subgroup (Figure 6(d)).

Next, we analysed the relationship between the CRSs and immune subtypes (C1–C4) in BLCA. The results showed that C1 (wound healing) and C2 (IFN-*γ* dominant) exhibited higher CRSs than C3 (inflammatory) and C4 (lymphocyte depleted) (Figure 6(e)). The CRS was positively correlated with the immune and stromal scores. Thus, patients in the high-CRS subgroup exhibited higher immune and stromal cell scores (Figures 6(f) and 6(g)).

### 3.6. Correlation between CRS Stratification and Drug Prediction

**Figure 7:**
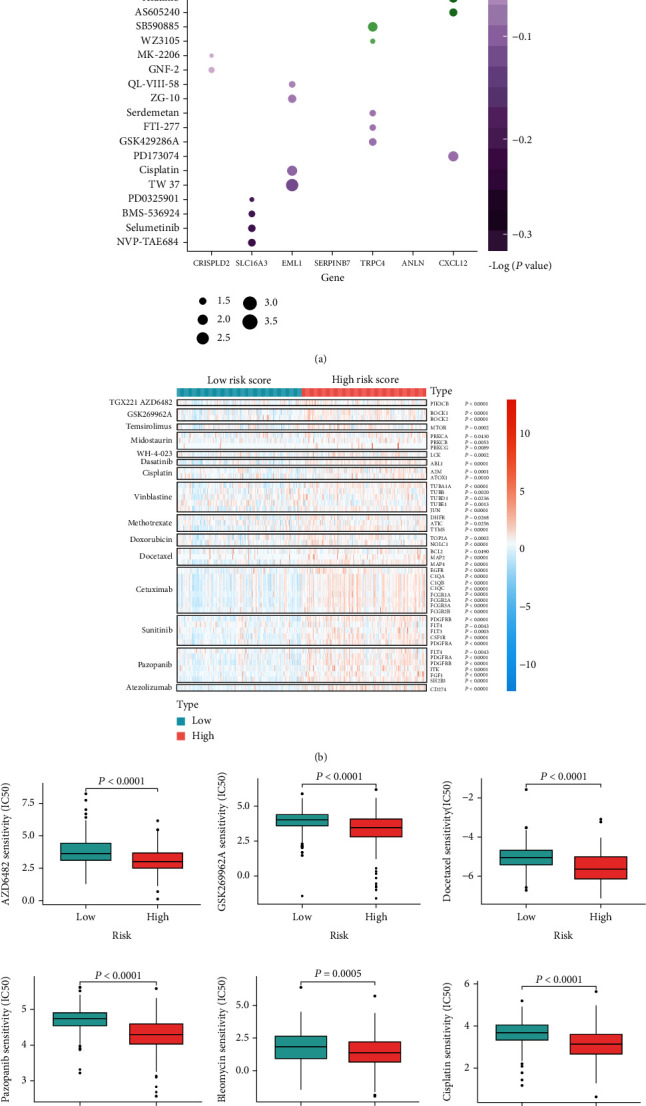
The CRS stratification and drug prediction. (a) Bubble diagram shows the correlation between IC50 of compounds (GDSC) and mRNA levels of seven CSR genes involved in the CRS model. (b) Heat map shows mRNA levels of targets whose compounds were screened from GDSC and common drugs between the high- and low-CRS subgroups. (c) pRRophetic predicts the different sensitivities of drugs between the high- and low-CRS subgroups. CRS: fibroblast common serum response risk score.

To investigate whether the CSR genes have the value in guiding clinical therapy in BLCA, we analysed the correlations between the CSR gene expression and the half-maximal inhibitory concentration (IC50) of drugs listed on the GDSC database. Surprisingly, we found that the respective responses of AZD6482 and TGX221 (both inhibitors of PI3K*β*) were positively associated with the high-level expression of most of the CSR genes. The *ANLN* and *SERPINB7* expression levels were revealed to have significant synergistic effects with docetaxel, a chemotherapeutic drug commonly used in clinical practice. The *SLC16A3* expression level had a strong synergistic effect with dasatinib (an inhibitor of Abl, Src, and c-Kit), tanespimycin (17-AAG, an inhibitor that targets heat shock protein 90), refametinib (a highly selective mitogen-activated protein kinase kinase (MEK) 1 and MEK2 inhibitor), and trametinib (a potent MEK1 and MEK2 inhibitor) (Figure 7(a)).

Subsequently, we performed variation analyses of the expression of targets of common drugs and compounds that were screened from the GDSC database in the high- and low-CRS subgroups. The targets of the chemotherapy drugs, namely, AZD6482, TGX221, GSK269962A, temsirolimus, midostaurin, WH-4-023, dasatinib, cisplatin, vinblastine, methotrexate, doxorubicin, docetaxel, sunitinib, and pazopanib, were observably upregulated in the high-CRS subgroup (Figure 7(b)). Moreover, the targets of cetuximab (viz., epidermal growth factor receptor, complement C1q A chain (C1QA), C1QB, C1QC, Fc gamma receptor (FCGR) 1A, FCGR2A, FCGR3A, and FCGR2B) and atezolizumab (viz., CD274) were also highly expressed in the high-CRS subgroup, suggesting that patients in this subgroup were more responsive to these chemotherapeutic and targeted drugs (Figure 7(b)). Through analysis of drug sensitivity by pRRophetic, we also inferred that patients with BLCA in the high-CRS subgroup would be more responsive to AZD6482, GSK269962A, docetaxel, pazopanib, bleomycin, cisplatin, dasatinib, midostaurin, and doxorubicin, than those patients in the low-CRS subgroup (Figure 7(c)).

### 3.7. Relationship of *ANLN* to Immunity in Bladder Cancer

**Figure 8:**
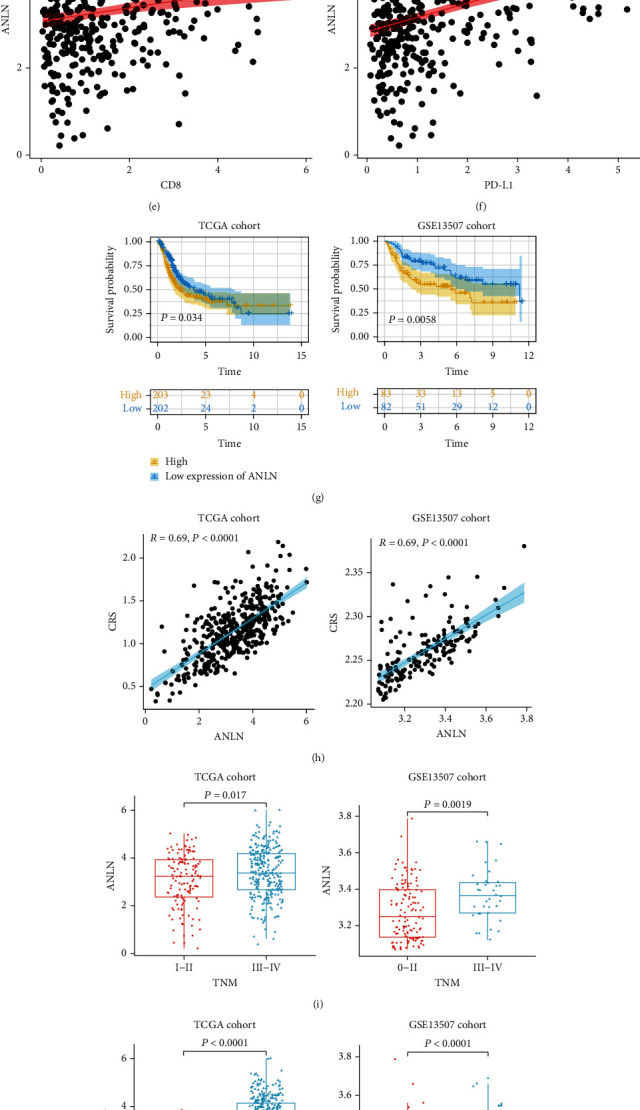
*ANLN* to immunity in bladder cancer. (a) Boxplot of the abundance of PD-L1 expression in the CRS groups in the TCGA-BLCA cohort. (b) Correlation analysis between PD-L1 expression and the CRS in TCGA cohort. mRNA levels of (c) ANLN or (d) EML1 in anti-PD-L1 responsiveness in the IMvigor210 cohort. (e, f) Correlation analyses of expression of ANLN and CD8 and PD-L1 in TCGA cohort. (g) Survival probability of patients with differential expression of ANLN of TCGA and GSE13507 cohorts. Levels of ANLN were identified according to the median of ANLN. (h) Correlation analyses of ANLN expression and the CRS. (i) Correlation analyses of expression of ANLN and TNM staging. (j) Correlation analyses of expression of ANLN and grade. Correlation analyses between (k) number of CD8+ T cells or (l) PD-L1 expression and ANLN expression of patients with BLCA in our hospital cohort. (m) Representative IHC or HE images of ANLN, CD8, PD-L1, and Ki67. CR: complete response; PR: partial response; SD: stable disease; PD: progressive disease; CRS: fibroblast common serum response risk score.

We further analysed the relationship between PD-L1 expression and the CRS and found that these two variables were positively correlated in TCGA cohort (Figures 8(a) and 8(b)). We also analysed the relationship between the CRS or CSR gene expression and the response to PD-L1-targeted treatment in the IMvigor210 cohort, whereupon patients showing a high level of *ANLN* or *EML1* expression were found to be more likely to benefit from anti-PD-L1 immune checkpoint treatment (Figures 8(c) and 8(d), Supplementary Figure 1A). Furthermore, *ANLN* expression was positively associated with *CD8* and *PD-L1* expression (Figures 8(e) and 8(f)), whereas *EML1* expression was not (Supplementary Figure 1B1). In the prognostic analysis, patients with a high *ANLN* expression level were found to have a worse prognosis in both TCGA (*P* = 0.0340) and GSE13507 (*P* = 0.0058) cohorts (Figure 8(g)). Moreover, *ANLN* expression was strongly positively correlated with the CRS in both TCGA (*R* = 0.69, *P* < 0.0001) and GSE13507 (*R* = 0.69, *P* < 0.0001) cohorts (Figure 8(h)). Among the seven CSR genes involved in the model, *ANLN* demonstrated good value in predicting the risk score (AUC = 0.9636, *P* < 0.0001; Supplementary Figure 2A). According to the DepMap database of 29 BLCA cell lines, *ANLN* had the lowest gene effect value of the seven CSR genes, suggesting that BLCA cells were highly dependent on this gene (Supplementary Figure 2B). Furthermore, patients with a high TNM stage (TNM III–IV) or a high tumour grade showed high levels of *ANLN* expression (Figures 8(i) and 8(j)), indicating that this gene can be used as an indicator of the malignant phenotype. For the tissue samples from our hospital cohort, IHC analysis was conducted to evaluate the relationship between ANLN protein levels and CD8+ cell infiltration or PD-L1 expression. The results indicated that patients expressing a high level of ANLN protein may have greater CD8+ cell infiltration and a high PD-L1 expression level (Figures 8(k)–8(m)). These findings suggest that ANLN can be an indicator of the malignant phenotype, disease prognosis, immune infiltration, and PD-L1 expression, which provides a rationale for its use in predicting the responses of patients with BLCA to anti-PD-L1 drugs.

## 4. Discussion

With the advances made in next-generation sequencing, more molecular mechanisms in cancer have been discovered [19]. In this study, we analysed the variations in oncogenic signature gene sets between the normal and BLCA tissues listed on TCGA. We found that the role of CSR-related signatures ranked first in BLCA. Through further analysis, we screened out the important genes affecting the prognosis of BLCA and use them to build the risk score stratification model. Using the LASSO algorithm, we obtained the CRSs, which can predict the prognosis of BLCA and the risk stratification for afflicted patients. The correlation analyses indicated that the CRS stratification could predict the responses to chemotherapy and immunotherapy. Furthermore, we found that *ANLN* was associated with the immune cell activity in the BLCA tumour microenvironment. Moreover, *ANLN* was related to the infiltration of CD8^+^ cell and PD-L1 expression in BLCA and could thus act as an indicator to predict the reactivity of the cancer to PD-L1 inhibitors.

The CRS model was based on seven genes: namely, *CLCX12*, *SERPINB7*, *CRISPLD2*, *EML1*, *TRPC4*, *SLC16A3*, and *ANLN*. To the best of our knowledge, few studies have evaluated the role of *SERPINB7*, *CRISPLD2*, *EML1*, and *TRPC4* in BLCA. As one of the most widely studied chemokines, CXCL12 has been reported in both haematological and solid tumours, including acute myeloid leukaemia [20], lymphoma [21], cervical cancer [22], lung cancer [23], colorectal cancer [24], and breast cancer [25]. It is mainly involved in the formation of tumour blood vessels, tumour growth, and metastasis. Moreover, CXCL12 also affects the tumour microenvironment, and its combination with IL-6 can mediate the homing and proliferation of tumour cells [26]. Du et al. found that CXCL12 and inflammatory fibroblasts act to regulate the tumour microenvironment in BLCA and can thus affect the outcome and efficacy of immunotherapy, further supporting the important role of CXCL12 in this disease [27].

Cancer cells continually reprogram metabolism in response to disease progression [28]. The Warburg effect shows that tumour cells are generally more dependent on glycolysis than on the increased use of oxidative phosphorylation [29]. The lactic acid molecules produced through glycolysis are transported via membrane monocarboxylic acid transporters to the tumour microenvironment where they regulate infiltrating immune cells, attenuating their antitumour immune response [30]. *SLC16A3*, which encodes MCT4 (an important member of the solute transport family 16), is widely expressed in tumour cells, immune cells, and astrocytes and depends on glycolysis for energy metabolism [31]. MCT4 mediates the efflux of lactic acid from tumour cells, which is essential for maintaining the cytoplasmic pH [32]. The protein is widely expressed in various urinary system tumours and is closely related to their prognoses. MCT4 expression is elevated in kidney and prostate cancers, and its high level indicates a poor prognosis and insensitivity to chemotherapy [33, 34]. Previous studies have indicated that MCT4 expression is upregulated in patients with BLCA and that individuals with high MCT4 expression levels have poor outcomes, which is in line with our findings [35]. Furthermore, the inhibition of MCT4 expression was found to lower the viability of BLCA cells [36]. These results suggest that MCT4 not only serves as an important molecular marker for diagnosis but is also a potential therapeutic target in BLCA.

*ANLN* encodes an anillin protein, a highly conserved actin-binding protein. It was found that this protein is closely associated with the tumourigenesis and tumour development, and its expression level affects the disease prognosis [37]. Research has shown that the ANLN protein level is increased in BLCA and is associated with the tumour stage, grade, and prognosis [38]. However, the exact role of *ANLN* in BLCA development and progression and the underlying mechanisms involved remain unclear. Chen et al. found that *ANLN* may affect the proliferation of BLCA cells by inhibiting the c-Jun N-terminal kinase signalling pathway [39]. However, the role of *ANLN* as an immunomodulatory gene in tumours has rarely been studied. Luo et al. found that *ANLN* could serve as an immune prediction biomarker in lung adenocarcinoma, and there were significant differences in PD-L1 expression levels between the high- and low-CRS subgroups [40]. We observed that *ANLN* expression was positively associated with CD8+ cell infiltration and *PD-L1* expression. Moreover, patients with a high *ANLN* expression level were more responsive to PD-L1 inhibitors. These results suggest that *ANLN* expression may indicate immune invasion by BLCA cells and thus could provide guidance for the use of PD-L1 inhibitors.

In the drug prediction study, we found that the respective responses of AZD6482 and TGX221 (both inhibitors of PI3K*β*) were positively associated with the high-level expression of most of the CSR genes. Moreover, patients in the high-CRS subgroup may be significantly responsive to AZD6482 and TGX221. However, we have not found any literature on the use of PI3K*β* inhibitors in BLCA. Gene differential expression analysis also suggested a high level of *PIK3CB* expression in the high-CRS group, which provides a rationale or its application as a biomarker for the use of PI3K*β* inhibitors for some patients with BLCA. Moreover, we also created some possible chemotherapy regimens for patients in the high-CRS subgroup. Although there are reports about the use of mammalian target of rapamycin (mTOR) inhibitors in BLCA, they have had limited success in clinical practice because of the simultaneous activation of compensatory pathways [41]. Our study provides an accurate guide for the use of mTOR inhibitors in patients with BLCA tumours. However, further research is needed to confirm our drug sensitivity findings.

## 5. Conclusions

Our study revealed the crucial role of the CSR signature in BLCA. Both CSR-related genes and CRS stratification hold the value in predicting the malignant phenotype, therapeutic efficacy of chemotherapeutic agents, immune invasion, and prognosis of BLCA. Of note, *ANLN* gene expression could not only act as a marker to predict the outcome of patients with BLCA but also improve their responses to immunotherapy.

## Data Availability

All data in our study are available upon request.

## Supplementary Materials

Supplementary 1 Supplementary Figure 1: CSR genes and tumour microenvironment in BLCA. (A) The CRS and mRNA levels of CSR genes in anti-PD-L1 responsiveness in the IMvigor210 cohort. (B) Correlation analyses of EML1 expression and CD8 and PD-L1 in the TCGA-BLCA cohort. CRS: fibroblast common serum response risk score.

Supplementary 2 Supplementary Figure 2: *ANLN* can well predict the CRS and may be dependent in BLCA cell lines. (A) ROC curves show the accuracy of CSR genes to predict the CRS. (B) Box plot shows gene effect of seven CRS genes in DepMap database for 29 BLCA cell lines. CRS: fibroblast common serum response risk score.

Supplementary 3 Supplementary Table 1: the oncogenic signature gene sets variation in bladder cancer.

Supplementary 4 Supplementary Table 2: differentially expressed fibroblast common serum response-related genes in bladder cancer.

Supplementary 5 Supplementary Table 3: Cox regression analysis screened fibroblast common serum response-related genes associated with survival in bladder cancer.

Supplementary 6 Supplementary Table 4: the CRS and risk stratification of TCGA cohort. CRS: fibroblast common serum response risk score; TCGA, the cancer genome atlas.

Supplementary 7 Supplementary Table 5: the CRS and risk stratification of GSE13507 cohort. CRS: fibroblast common serum response risk score.

Supplementary 8 Supplementary Table 6: genes involved in cancer signalling pathways.

Supplementary 9 Supplementary Table 7: genes involved in characteristic pathway in bladder cancer.
