# Supplementary material for: Fibroblast Common Serum Response Signature-Related Classification Affects the Tumour Microenvironment and Predicts Prognosis in Bladder Cancer
Source: Oxid Med Cell Longev. 2022 Oct 19;2022:5645944. doi: 10.1155/2022/5645944 (PMC9606836; doi:10.1155/2022/5645944)
Supplement: Supplementary 1 — Supplementary Figure 1: CSR genes and tumour microenvironment in BLCA. (A) The CRS and mRNA levels of CSR genes in anti-PD-L1 responsiveness in the IMvigor210 cohort. (B) Correlation analyses of EML1 expression and CD8 and PD-L1 in the TCGA-BLCA cohort. CRS: fibroblast common serum response risk score. [file 5645944.f1.pdf]

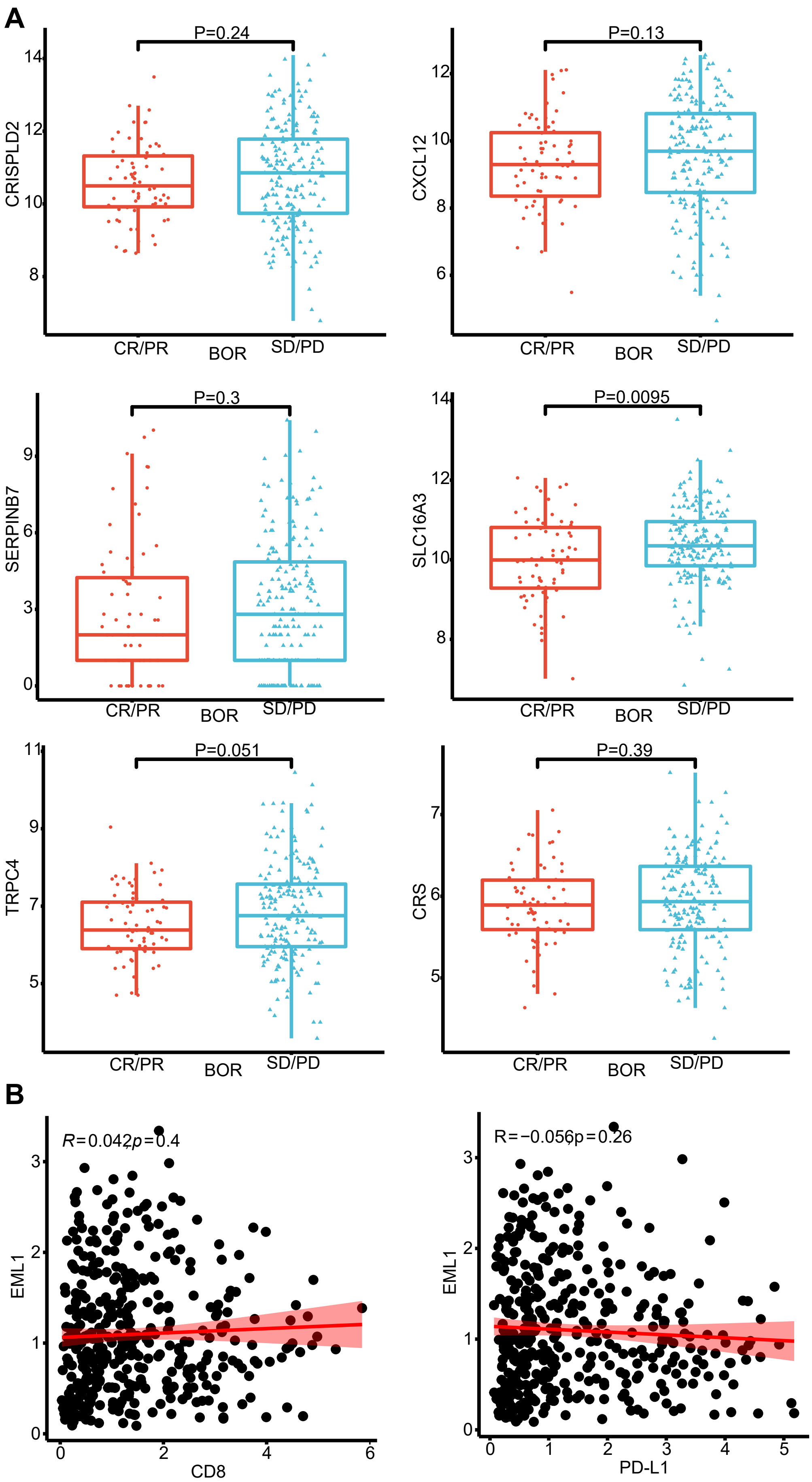

Supplementary Figure 1. CSR genes and tumour microenvironment in BLCA.
(A) The CRS and mRNA levels of CSR genes in anti-PD-L1 responsiveness in the IMvigor210 cohort. (B) Correlation analyses of EML1expression and CD8 and PD-L1 in the TCGA-BLCA cohort. CRS, fibroblast common serum response risk score.
