# Supplementary material for: Fibroblast Common Serum Response Signature-Related Classification Affects the Tumour Microenvironment and Predicts Prognosis in Bladder Cancer
Source: Oxid Med Cell Longev. 2022 Oct 19;2022:5645944. doi: 10.1155/2022/5645944 (PMC9606836; doi:10.1155/2022/5645944)
Supplement: Supplementary 2 — Supplementary Figure 2: ANLN can well predict the CRS and may be dependent in BLCA cell lines. (A) ROC curves show the accuracy of CSR genes to predict the CRS. (B) Box plot shows gene effect of seven CRS genes in DepMap database for 29 BLCA cell lines. CRS: fibroblast common serum response risk score. [file 5645944.f2.pdf]

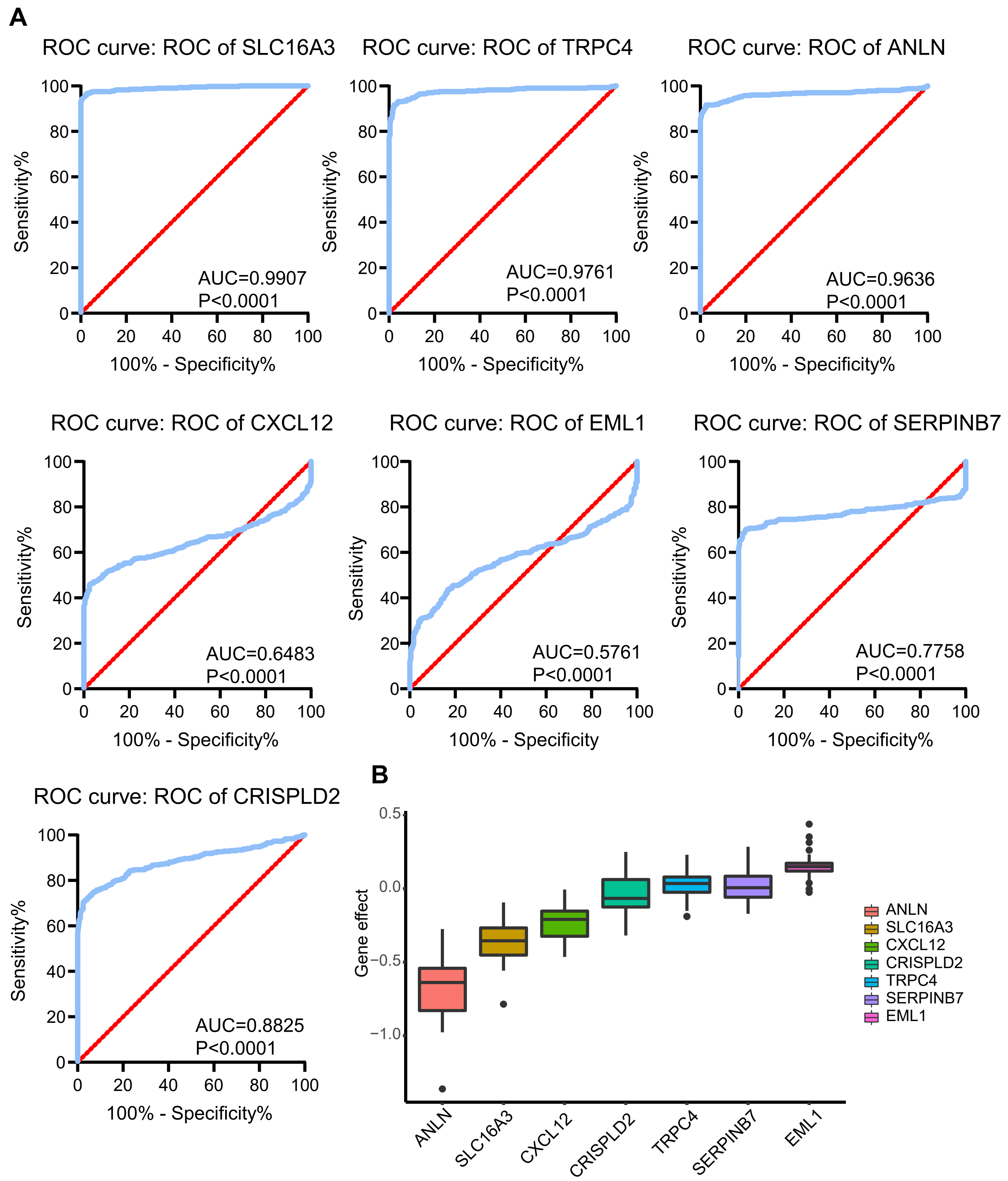

Supplementary Figure 2. *ANLN* can well predict the CRS and may be dependent in BLCA cell lines. (A) ROC curves show the accuracy of CSR genes to predict the CRS. (B) Box plot shows gene effect of seven CRS genes in Depmap database for 29 BLCA cell lines. CRS, fibroblast common serum response risk score.
