# Supplementary material for: Fibroblast Common Serum Response Signature-Related Classification Affects the Tumour Microenvironment and Predicts Prognosis in Bladder Cancer
Source: Oxid Med Cell Longev. 2022 Oct 19;2022:5645944. doi: 10.1155/2022/5645944 (PMC9606836; doi:10.1155/2022/5645944)
Supplement: Supplementary 3 — Supplementary Table 1: the oncogenic signature gene sets variation in bladder cancer. [file 5645944.f3.pdf]

Supplementary Table 1.The oncogenic signature gene sets variation in bladder cancer.

oncogenic signature gene set	log(Fold change)	Average expression	adjusted P-value
KRAS.KIDNEY_UP.V1_UP	-0.434815459	-0.030994483	2.33E-14
P53_DN.V1_DN	-0.283971842	-0.032412633	1.36E-10
MEL18_DN.V1_DN	-0.273652065	-0.029711682	5.22E-13
LEF1_UP.V1_UP	-0.265768495	-0.037837549	2.67E-07
KRAS.PROSTATE_UP.V1_DN	-0.242536479	-0.021598029	4.54E-12
ATF2_UP.V1_DN	-0.230429947	-0.038921833	1.75E-11
BMI1_DN_MEL18_DN.V1_DN	-0.221249589	-0.031202065	9.62E-10
RPS14_DN.V1_UP	-0.202235026	-0.032512296	0.002985664
PTEN_DN.V1_UP	-0.201956476	-0.023909196	8.78E-09
STK33_UP	-0.200466273	-0.040980658	3.77E-06
BMI1_DN.V1_DN	-0.200122236	-0.028213332	1.06E-09
KRAS.300_UP.V1_UP	-0.189456486	-0.01168936	1.36E-08
STK33_NOMO_UP	-0.187763136	-0.041838241	1.47E-05
RAF_UP.V1_DN	-0.186564624	-0.047611295	6.30E-07
HINATA_NFKB_IMMU_INF	-0.181572031	-0.060662947	0.035487553
KRAS.600_UP.V1_UP	-0.180715361	-0.017003891	9.32E-09
PTEN_DN.V2_UP	-0.177596187	-0.03337012	6.30E-07
CAHOY_ASTROGLIAL	-0.173517401	-0.037497732	0.00066383
TGFB_UP.V1_UP	-0.168300483	-0.043821446	3.47E-05
AKT_UP.V1_DN	-0.156197612	-0.042911609	4.31E-05
CSR_LATE_UP.V1_DN	-0.155401771	-0.059708355	9.57E-08
ATF2_S_UP.V1_DN	-0.150243898	-0.041495155	9.31E-05
ESC_V6.5_UP_EARLY.V1_DN	-0.144301093	-0.031835328	0.004961332
PRC2_EZH2_UP.V1_UP	-0.143029825	-0.043209359	3.77E-06
BCAT_GDS748_UP	-0.140306645	-0.052813294	0.000366483
CAMP_UP.V1_DN	-0.139386474	-0.043762938	2.58E-05
STK33_SKM_UP	-0.13705969	-0.037666091	0.000246232
E2F1_UP.V1_DN	-0.130209079	-0.043355917	1.55E-05
KRAS.BREAST_UP.V1_DN	-0.130028887	-0.01203767	0.000344798
MTOR_UP.V1_DN	-0.12858527	-0.041899622	8.10E-06
CRX_DN.V1_DN	-0.124639803	-0.032756821	0.000271295
IL21_UP.V1_DN	-0.12321085	-0.016505598	3.66E-05
KRAS.BREAST_UP.V1_UP	-0.122551913	-0.007161008	0.000558159
BCAT.100_UP.V1_UP	-0.122306109	-0.041349318	0.004991902
IL15_UP.V1_DN	-0.119373921	-0.02169249	0.000146068
BMI1_DN_MEL18_DN.V1_UP	-0.117902747	-0.052950826	0.055302483
PTEN_DN.V1_DN	-0.11590619	-0.025077128	0.000264624
BRCA1_DN.V1_UP	-0.115153967	-0.001742385	0.000682048
HOXA9_DN.V1_UP	-0.113511861	-0.04404954	0.009093489
JAK2_DN.V1_UP	-0.111928763	-0.013427342	3.00E-05
ATM_DN.V1_DN	-0.109712952	-0.012491386	0.000244262
CAHOY_NEURONAL	-0.10930614	0.000890784	0.009974453
CYCLIN_D1_KE_.V1_DN	-0.104310659	-0.008625848	6.03E-06
RAF_UP.V1_UP	-0.103670423	-0.04538917	0.005823952
EGFR_UP.V1_UP	-0.102374308	-0.04374092	0.026546962
KRAS.600.LUNG.BREAST_UP.V1_UP	-0.101422439	-0.0160597	0.001279389
EIF4E_DN	-0.100068073	-0.031534667	0.079611077
SNF5_DN.V1_DN	-0.09979313	-0.02348148	0.000591887
KRAS.600.LUNG.BREAST_UP.V1_DN	-0.095447467	-0.017628267	0.000496585

VEGF_A_UP.V1_UP	-0.092034298	-0.033726199	0.022477411
IL2_UP.V1_DN	-0.090285093	-0.025672808	0.023925364
KRAS.LUNG.BREAST_UP.V1_DN	-0.086610711	-0.016141578	0.011978991
RELA_DN.V1_DN	-0.085619291	-0.010395216	0.007667923
NOTCH_DN.V1_DN	-0.084219702	-0.027604133	0.001279389
KRAS.PROSTATE_UP.V1_UP	-0.082069806	-0.018152615	0.003549884
RELA_DN.V1_UP	-0.081680854	-0.03463779	0.003620517
KRAS.300_UP.V1_DN	-0.081339786	-0.011728239	0.017869268
KRAS.LUNG.BREAST_UP.V1_UP	-0.079147517	-0.022221914	0.04233301
CTIP_DN.V1_DN	-0.075823848	-0.019330987	0.002639267
LTE2_UP.V1_UP	-0.074093123	-0.042122677	0.039524472
CAHOY_OLIGODENDROCUTIC	-0.071272979	-0.046512908	0.0065118
MTOR_UP.N4.V1_DN	-0.070068791	-0.036254727	0.255030656
P53_DN.V2_UP	-0.068788854	-0.01953235	0.050508424
JNK_DN.V1_UP	-0.065371307	-0.032377526	0.014126895
ESC_J1_UP_LATE.V1_UP	-0.064477092	-0.023197304	0.082295217
CTIP_DN.V1_UP	-0.063269214	-0.023147195	0.10329293
KRAS.600_UP.V1_DN	-0.060704109	-0.01399074	0.032313636
CORDENONSI_YAP_CONSERVED_SIGNA	-0.059003038	-0.049576953	0.38246718
KRAS.50_UP.V1_UP	-0.058976567	0.000195307	0.253150405
RAPA_EARLY_UP.V1_UP	-0.058108222	-0.025784081	0.016009499
KRAS.LUNG_UP.V1_UP	-0.047904462	-0.028017034	0.16886016
KRAS.50_UP.V1_DN	-0.047445035	-0.004701503	0.358661644
BCAT.100_UP.V1_DN	-0.045731497	-0.026026306	0.3108811
P53_DN.V1_UP	-0.039112013	-0.039653852	0.271570181
PRC2_SUZ12_UP.V1_UP	-0.037450382	-0.027241671	0.092966312
PDGF_UP.V1_DN	-0.035873526	-0.017206829	0.223277799
LEF1_UP.V1_DN	-0.034493243	-0.044170556	0.512802915
ESC_V6.5_UP_LATE.V1_UP	-0.033057239	-0.036491077	0.378877762
ERBB2_UP.V1_UP	-0.033044782	-0.044012358	0.512802915
PRC1_BMI_UP.V1_UP	-0.031052922	-0.021001742	0.212162076
STK33_SKM_DN	-0.029649097	-0.03870008	0.227595004
WNT_UP.V1_DN	-0.028382108	-0.030767388	0.235982782
IL21_UP.V1_UP	-0.027807438	-0.028809444	0.298895683
KRAS.LUNG_UP.V1_DN	-0.027299067	-0.006490336	0.512802915
JNK_DN.V1_DN	-0.024927947	-0.027010557	0.413088675
NOTCH_DN.V1_UP	-0.023980976	-0.016414926	0.335693763
ATM_DN.V1_UP	-0.022792609	-0.016864475	0.498900479
CAHOY_ASTROCYTIC	-0.018379608	-0.026158773	0.526339454
PGF_UP.V1_UP	-0.017799915	-0.024376042	0.806192758
KRAS.AMP.LUNG_UP.V1_UP	-0.016644172	-0.017506645	0.721944156
MEK_UP.V1_UP	-0.015116248	-0.039522683	0.784107211
GLI1_UP.V1_UP	-0.013127369	-0.048904235	0.819881024
BCAT_BILD_ET_AL_DN	-0.010942367	-0.032147672	0.895967405
BMI1_DN.V1_UP	-0.010923959	-0.048751272	0.881880952
PRC2_EED_DN.V1_UP	-0.0086087	-0.019677521	0.786250863
PKCA_DN.V1_DN	-0.005572581	-0.023015115	0.789755794
IL2_UP.V1_UP	-0.003879519	-0.051894166	0.925771529
NRL_DN.V1_DN	-0.001827225	-0.041543879	0.936127792
P53_DN.V2_DN	-7.38E-06	-0.025945602	0.999702784
KRAS.AMP.LUNG_UP.V1_DN	0.001808217	-0.027486211	0.960337746

PGF_UP.V1_DN	0.003187567	-0.027667908	0.929331937
ALK_DN.V1_DN	0.003249754	-0.01689365	0.929331937
TGFB_UP.V1_DN	0.003627831	-0.041150918	0.928072865
PKCA_DN.V1_UP	0.003901648	-0.006747944	0.925771529
KRAS.DF.V1_UP	0.003918677	-0.055589278	0.920998659
KRAS.KIDNEY_UP.V1_DN	0.004262541	-0.01337614	0.928005077
KRAS.DF.V1_DN	0.007479618	-0.021177806	0.819881024
ESC_J1_UP_EARLY.V1_UP	0.007655513	-0.039144886	0.806192758
MEL18_DN.V1_UP	0.010982244	-0.051326272	0.881880952
CRX_NRL_DN.V1_DN	0.013765434	-0.037730767	0.654205519
MYC_UP.V1_DN	0.014000605	-0.036137503	0.701972994
ESC_V6.5_UP_EARLY.V1_UP	0.024356423	-0.028692513	0.512802915
NRL_DN.V1_UP	0.025802822	-0.031517556	0.460354975
PDGF_UP.V1_UP	0.030715295	-0.034201532	0.378877762
CSR_EARLY_UP.V1_DN	0.031290997	-0.043993571	0.603861903
CRX_NRL_DN.V1_UP	0.033704935	-0.038789034	0.332089263
CRX_DN.V1_UP	0.033868175	-0.030208498	0.314381375
PRC1_BMI_UP.V1_DN	0.03498154	-0.010169928	0.202534703
TBK1.DF_UP	0.036495788	-0.042720361	0.22857957
GCNP_SHH_UP_EARLY.V1_DN	0.040341101	-0.037274543	0.145852488
LTE2_UP.V1_DN	0.043255586	-0.040024176	0.366753855
AKT_UP_MTOR_DN.V1_DN	0.043313956	-0.034729713	0.092966312
ESC_J1_UP_EARLY.V1_DN	0.043943857	-0.043639591	0.07906491
STK33_NOMO_DN	0.048574613	-0.035021407	0.06395069
WNT_UP.V1_UP	0.052653852	-0.029077732	0.039038564
CYCLIN_D1_UP.V1_DN	0.054106669	-0.018441317	0.03230589
PRC2_SUZ12_UP.V1_DN	0.056455718	-0.016795172	0.05266259
MEK_UP.V1_DN	0.060842117	-0.033269069	0.133819218
RAPA_EARLY_UP.V1_DN	0.061680387	-0.02145606	0.03162099
IL15_UP.V1_UP	0.062834023	-0.042667977	0.032934339
STK33_DN	0.066244023	-0.037749824	0.008770412
BRCA1_DN.V1_DN	0.066615478	-0.019918189	0.035926986
RB_P130_DN.V1_DN	0.06698231	-0.025154429	0.193086896
ATF2_S_UP.V1_UP	0.067213705	-0.02610519	0.003272202
SNF5_DN.V1_UP	0.068186632	-0.03926007	0.221953534
NFE2L2.V2	0.068262041	-0.013187029	0.012290996
CYCLIN_D1_UP.V1_UP	0.070609219	-0.025628722	0.021804443
JAK2_DN.V1_DN	0.07307467	-0.031874473	0.290678498
YAP1_UP	0.073623132	-0.040703285	0.039810733
AKT_UP_MTOR_DN.V1_UP	0.073924948	-0.030097373	0.013897071
RB_DN.V1_DN	0.076004231	-0.038004771	0.050671517
ALK_DN.V1_UP	0.080225247	-0.010307646	0.049192401
DCA_UP.V1_UP	0.081871569	-0.011422533	0.004057248
ESC_V6.5_UP_LATE.V1_DN	0.085412531	-0.038655577	0.001078966
PDGF_ERK_DN.V1_DN	0.085796719	-0.037874896	0.021719463
EGFR_UP.V1_DN	0.088718108	-0.034128755	0.011053602
SINGH_KRAS_DEPENDENCY_SIGNATUF	0.090780306	-0.02967758	0.298895683
YAP1_DN	0.09423157	-0.01534857	0.041673246
PTEN_DN.V2_DN	0.098666469	-0.042514958	0.006958923
ESC_J1_UP_LATE.V1_DN	0.101435442	-0.037778154	0.00067149
PDGF_ERK_DN.V1_UP	0.105980934	-0.023717517	0.000150634

GCNP_SHH_UP_LATE.V1_DN	0.110599098	-0.027781029	1.63E-05
ERBB2_UP.V1_DN	0.118455131	-0.029601074	0.02693617
SRC_UP.V1_UP	0.119833911	-0.018822056	0.017869268
CYCLIN_D1_KE_.V1_UP	0.12101366	-0.031830602	1.03E-05
BCAT_BILD_ET_AL_UP	0.121406243	-0.038214323	0.009546
E2F3_UP.V1_UP	0.122835759	-0.045706853	0.006784565
ATF2_UP.V1_UP	0.124052052	-0.01979249	2.25E-05
AKT_UP.V1_UP	0.125559351	-0.029649208	0.000857122
SIRNA_EIF4GI_UP	0.13124409	-0.039508712	0.011246487
DCA_UP.V1_DN	0.133832972	-0.023838914	8.29E-05
CSR_EARLY_UP.V1_UP	0.134008185	-0.044761336	0.012206623
BCAT_GDS748_DN	0.148546272	-0.044576533	0.0012093
TBK1.DF_DN	0.150196354	-0.028957419	0.017985573
MTOR_UP.N4.V1_UP	0.155306757	-0.042728929	2.76E-05
TBK1.DN.48HRS_DN	0.166232204	-0.048981037	0.000138926
VEGF_A_UP.V1_DN	0.170077672	-0.028533355	0.003496521
E2F3_UP.V1_DN	0.180923652	-0.024414685	1.91E-07
SRC_UP.V1_DN	0.186399295	-0.026342946	9.62E-10
PRC2_EZH2_UP.V1_DN	0.188668148	-0.024770324	5.27E-06
TBK1.DN.48HRS_UP	0.192776916	-0.033025064	0.000545945
RB_P130_DN.V1_UP	0.196665374	-0.025182899	0.000219615
HINATA_NFKB_MATRIX	0.197729422	-0.038031168	0.012206623
HOXA9_DN.V1_DN	0.199681275	-0.035927603	1.67E-06
SIRNA_EIF4GI_DN	0.199815714	-0.022703417	8.34E-06
RB_P107_DN.V1_DN	0.200476416	-0.033293963	4.51E-06
MTOR_UP.V1_UP	0.208270868	-0.028240082	1.98E-08
GCNP_SHH_UP_EARLY.V1_UP	0.227104178	-0.017663041	2.70E-05
EIF4E_UP	0.245398443	-0.032922731	6.02E-06
GLI1_UP.V1_DN	0.249257809	-0.017718402	1.10E-05
CAMP_UP.V1_UP	0.252588839	-0.041809475	6.62E-09
RB_DN.V1_UP	0.262077714	-0.026781701	1.82E-08
E2F1_UP.V1_UP	0.281157407	-0.029179245	2.85E-07
PRC2_EED_DN.V1_DN	0.281647375	-0.034728755	7.14E-10
MYC_UP.V1_UP	0.282979842	-0.03502449	4.11E-06
GCNP_SHH_UP_LATE.V1_UP	0.285854249	-0.021013123	6.30E-07
RPS14_DN.V1_DN	0.321968495	-0.025737716	9.32E-09
RB_P107_DN.V1_UP	0.327731905	-0.025319635	8.32E-07
CSR_LATE_UP.V1_UP	0.412539312	-0.016441134	3.61E-09
