# Supplementary material for: Fibroblast Common Serum Response Signature-Related Classification Affects the Tumour Microenvironment and Predicts Prognosis in Bladder Cancer
Source: Oxid Med Cell Longev. 2022 Oct 19;2022:5645944. doi: 10.1155/2022/5645944 (PMC9606836; doi:10.1155/2022/5645944)
Supplement: Supplementary 4 — Supplementary Table 2: differentially expressed fibroblast common serum response-related genes in bladder cancer. [file 5645944.f4.pdf]

Supplementary Table 2.Differentially expressed fibroblast common serum response-related genes in bladder cancer.

Gene	Normal Mean	Tumour Mean	log(Fold change)	P-value
TCF19	4.438566105	14.52887183	1.710757093	7.34E-08
CXCL12	22.21386595	5.519573233	-2.008831862	1.44E-09
FEN1	8.263308105	26.48063435	1.680146317	7.95E-09
GINS3	1.025037216	2.315217731	1.175471585	1.90E-06
ZWILCH	2.075238274	5.126074169	1.30457736	1.03E-09
MT1H	0.204929847	1.863326867	3.184678751	9.06E-05
UHRF1	0.960100658	5.723098047	2.575538751	7.19E-11
TRIP13	1.985285484	9.708573465	2.289912846	3.39E-09
MCM3	23.29222316	50.54089283	1.117602804	6.19E-08
SERPINB7	0.227546789	2.377609958	3.385276933	0.0035165
S1PR1	14.48346442	4.428334294	-1.709570694	1.32E-08
CRISPLD2	30.79418305	8.024750014	-1.9401295	6.47E-09
FANCA	0.532123831	2.513869507	2.240075842	4.96E-11
PAICS	9.327434526	21.02520348	1.172567529	1.62E-09
BIRC5	4.557025096	19.46419005	2.094658091	9.21E-09
IGFBP3	92.78345053	263.7140336	1.507034942	0.00444699
SHCBP1	1.314102524	4.192189543	1.673626111	1.52E-07
TRPC3	0.930772305	0.166685016	-2.481303866	2.69E-09
EML1	6.101148047	1.41489028	-2.108390558	3.45E-05
PRIM1	1.856003989	4.932081852	1.409996931	1.23E-07
TUBG1	12.98838068	29.87591316	1.201761234	6.59E-10
JPT1	25.37913253	72.40226347	1.512392042	7.95E-09
MRAS	7.111488116	2.979921912	-1.254876957	0.00325812
CDCA8	2.481830721	14.22263049	2.518711696	1.12E-10
CASP2	3.008891105	7.627920993	1.342058006	7.66E-11
KRT18	113.8938527	350.1713989	1.620371372	1.45E-05
CHEK2	2.376815063	4.875275454	1.03645408	1.70E-07
MYBL2	6.89751108	40.46534728	2.552539203	1.07E-09
NDC1	4.405048684	10.10184054	1.197388305	6.40E-09
CDC6	1.814868358	8.094323717	2.157045642	8.59E-10
FOXM1	2.660193311	13.51299827	2.344744824	3.16E-09
TRPC4	0.691063184	0.223582955	-1.628007417	2.21E-06
EEF1E1	3.628938105	7.803026938	1.10448643	1.20E-09
GREM2	3.173538553	0.407543966	-2.961064756	2.46E-11
CDK5	3.262429895	7.535281417	1.207714492	6.91E-10
SRM	26.83384632	54.03092716	1.009731576	6.55E-09
POLD1	3.348499263	9.835539854	1.554489591	1.27E-10
SLC16A3	7.940506342	19.7189487	1.312279726	0.00013161
NUF2	1.129838244	7.528649456	2.736274847	1.60E-10
TPM2	433.3469437	62.1031204	-2.802784865	3.11E-07
C19orf48	10.00082437	29.38556551	1.554988736	2.15E-10
RRM2	5.484550443	20.23330141	1.883286464	1.06E-07
RAD18	1.251314311	2.918931869	1.22199632	8.41E-09
MT1F	2.050927063	4.471039955	1.124334252	0.00683566
TEX30	2.425918211	5.821526947	1.262866701	9.87E-08
NMB	5.771886053	17.35729638	1.588427523	3.02E-07
ZWINT	4.222939026	22.85457288	2.436163535	7.28E-11
LMNB2	10.88510168	27.13742719	1.317929066	1.69E-08
CLSPN	0.449606511	1.896061511	2.076270935	8.52E-08

CCDC34	1.995948684	4.973129071	1.317079245	2.33E-08
ACTL6A	8.413060947	20.79633144	1.305626352	2.59E-10
DNAJB11	8.053431158	17.48763458	1.118659681	4.35E-10
KIF22	9.370071684	22.97051118	1.293650972	1.18E-08
ANLN	5.0148319	11.58042815	1.207415347	1.07E-07
UBE2C	10.44870126	70.6156068	2.756663438	5.29E-11
YEATS4	6.051172368	16.62017698	1.457649159	3.77E-08
EZH2	1.927299325	7.497377879	1.959805467	1.91E-09
SNRPA1	6.063971632	12.42259723	1.034631924	1.31E-09
KATNAL1	3.018680658	1.148034958	-1.394751571	0.00149336
CDC7	1.369904563	4.559157802	1.734691956	1.10E-08
DTYMK	7.039810316	16.78610661	1.253659187	1.42E-09
NETO2	1.319008155	3.41078105	1.370648662	0.00060194
CDK1	2.775738716	15.76485713	2.505768419	5.71E-11
PBK	1.997630364	7.282822465	1.866208021	2.01E-07
DCBLD1	1.4602859	3.808622369	1.383018397	6.67E-07
KISS1	0.894945776	9.238030451	3.367713123	0.00463593
CKAP2	3.778515005	10.02078202	1.407103844	8.61E-08
SSTR1	0.950985453	0.222556775	-2.095249854	1.26E-10
