# Supplementary material for: Fibroblast Common Serum Response Signature-Related Classification Affects the Tumour Microenvironment and Predicts Prognosis in Bladder Cancer
Source: Oxid Med Cell Longev. 2022 Oct 19;2022:5645944. doi: 10.1155/2022/5645944 (PMC9606836; doi:10.1155/2022/5645944)
Supplement: Supplementary 5 — Supplementary Table 3: Cox regression analysis screened fibroblast common serum response-related genes associated with survival in bladder cancer. [file 5645944.f5.pdf]

Supplementary Table 3. Cox regression analysis screened fibroblast common serum response-related genes associated with survival in bladder cancer.

Genes	Hazard ratio	HR.95L	HR.95H	P value
CXCL12	1.136578637	1.020482208	1.26588292	0.019870786
GMNN	0.784950906	0.655330619	0.940209274	0.008551258
SERPINB	1.177401066	1.059665881	1.308217331	0.002380816
CRISPLD	1.165207939	1.033726252	1.313413043	0.012316145
PSMD2	1.342071172	1.05294395	1.710589659	0.017467438
MT1X	1.113326252	1.022492352	1.212229452	0.013428515
FARSB	1.358847802	1.012588395	1.823512257	0.041020261
EML1	1.245038127	1.009872259	1.534966351	0.040175534
CTPS1	1.293219774	1.067537381	1.56661248	0.008591998
PDCL3	0.568362956	0.421126711	0.76707661	0.000221302
MAZ	1.307234558	1.036476	1.648723356	0.023665505
KCNC4	1.736705347	1.075996494	2.803118301	0.023831384
COPS3	1.442148092	1.125229453	1.848326236	0.003829526
TRPC4	1.733721788	1.015339459	2.9603806	0.043828399
TAGLN3	1.230555122	1.033781678	1.464783078	0.019612442
ALKBH7	0.730443336	0.580819656	0.918611243	0.007234133
SLC16A3	1.157235294	1.016476041	1.317486563	0.027319064
LTBP2	1.181551086	1.045212289	1.335674085	0.007656635
PRMT5	1.465196113	1.169442033	1.835746953	0.000898034
HAUS2	1.596011503	1.169172506	2.178679966	0.003236916
EIF3B	1.362737784	1.016777021	1.826412508	0.038329514
MTHFD1	1.294610572	1.001754233	1.673081558	0.048454688
ANLN	1.257665781	1.082096495	1.461721043	0.00280386
UTP18	1.359870868	1.002025735	1.845510264	0.048500937
KATNAL1	1.35861506	1.086609287	1.698710753	0.007173434
CDH2	1.177023642	1.029973802	1.345067855	0.016679433
HYPK	0.361907381	0.183207094	0.714912015	0.003431788
