# Supplementary material for: Fibroblast Common Serum Response Signature-Related Classification Affects the Tumour Microenvironment and Predicts Prognosis in Bladder Cancer
Source: Oxid Med Cell Longev. 2022 Oct 19;2022:5645944. doi: 10.1155/2022/5645944 (PMC9606836; doi:10.1155/2022/5645944)
Supplement: Supplementary 6 — Supplementary Table 4: the CRS and risk stratification of TCGA cohort. CRS: fibroblast common serum response risk score; TCGA, the cancer genome atlas. [file 5645944.f6.pdf]

Supplementary Table 4. The CRS and risk stratification of TCGA cohort.

Patient ID	CRS	Risk stratification
TCGA-BL-A13J	1.316458619	high
TCGA-FD-A3SM	1.228987298	high
TCGA-K4-A4AC	1.064752678	low
TCGA-2F-A9KR	0.900723462	low
TCGA-XF-A9SY	1.656338985	high
TCGA-FD-A5BT	1.305793117	high
TCGA-YC-A8S6	1.41322738	high
TCGA-2F-A9KW	1.320758147	high
TCGA-FD-A6TG	1.251509609	high
TCGA-XF-AAMR	1.327080586	high
TCGA-FD-A5BZ	1.599386641	high
TCGA-DK-A3IM	1.269326529	high
TCGA-G2-A2EL	1.088439956	low
TCGA-CU-A72E	1.090667991	low
TCGA-ZF-AA4X	1.087962172	low
TCGA-4Z-AA7S	1.106433821	low
TCGA-DK-A2I4	1.431351441	high
TCGA-4Z-AA89	0.732322143	low
TCGA-KQ-A41O	0.730303292	low
TCGA-E7-A7XN	0.958591454	low
TCGA-XF-A9SM	1.416311092	high
TCGA-DK-A3IS	0.985308945	low
TCGA-DK-AA6T	0.764030034	low
TCGA-XF-AAN0	1.300649179	high
TCGA-GC-A3YS	1.205504382	high
TCGA-CF-A47S	0.418087388	low
TCGA-E7-A8O8	0.657814157	low
TCGA-KQ-A41R	0.747641284	low
TCGA-DK-AA6W	1.330341497	high
TCGA-E7-A6MF	0.401894698	low
TCGA-GC-A3I6	1.290702314	high
TCGA-DK-A2I1	1.026844491	low
TCGA-DK-A3IQ	1.677091637	high
TCGA-K4-AAQO	1.386613634	high
TCGA-ZF-A9R1	1.0431954	low
TCGA-GD-A3OP	1.09884957	low
TCGA-XF-AAN8	1.198309417	high
TCGA-4Z-AA82	1.558770005	high
TCGA-FD-A3SS	1.860583222	high
TCGA-BT-A0YX	1.12032731	low
TCGA-DK-A3IN	1.398996318	high
TCGA-DK-A3WW	1.242981759	high
TCGA-ZF-AA4N	1.635500385	high
TCGA-FJ-A871	1.177802971	high
TCGA-CF-A1HR	1.076337926	low
TCGA-BT-A20U	1.467704091	high
TCGA-ZF-AA5P	1.284830847	high

TCGA-G2-AA3D	1.15508993	high
TCGA-ZF-AA54	1.492456027	high
TCGA-CU-A0YR	1.210743588	high
TCGA-GC-A3OO	1.286918221	high
TCGA-XF-A9SW	1.713173555	high
TCGA-BT-A20P	0.957984807	low
TCGA-FD-A6TA	1.043171149	low
TCGA-GV-A6ZA	0.870048242	low
TCGA-GV-A3QH	1.251812139	high
TCGA-CF-A5UA	0.644927033	low
TCGA-H4-A2HO	0.628730496	low
TCGA-BL-A0C8	1.268066591	high
TCGA-SY-A9G5	1.345583188	high
TCGA-4Z-AA7O	1.079662746	low
TCGA-FD-A3B5	1.581594421	high
TCGA-BT-A20X	1.512398578	high
TCGA-CF-A47T	0.681449606	low
TCGA-GV-A3JZ	1.272885971	high
TCGA-GC-A3BM	1.059240665	low
TCGA-UY-A78N	1.086313488	low
TCGA-4Z-AA83	0.873365733	low
TCGA-DK-A3WY	1.211876303	high
TCGA-FD-A5BY	1.569790689	high
TCGA-E7-A5KE	1.127350115	low
TCGA-DK-AA6U	0.939046503	low
TCGA-FD-A43X	0.738045107	low
TCGA-FD-A5C0	1.187600353	high
TCGA-E7-A85H	1.019526689	low
TCGA-GC-A4ZW	1.140063978	low
TCGA-GU-AATP	1.117451934	low
TCGA-DK-A6B5	1.093279353	low
TCGA-GD-A3OQ	1.366858909	high
TCGA-G2-A3IE	0.5153214	low
TCGA-YC-A9TC	1.222070805	high
TCGA-2F-A9KQ	1.070518033	low
TCGA-UY-A8OB	1.314739138	high
TCGA-LC-A66R	1.335338139	high
TCGA-G2-AA3F	0.949112492	low
TCGA-K4-A6FZ	1.380419282	high
TCGA-UY-A9PA	0.909570484	low
TCGA-5N-A9KI	1.262258448	high
TCGA-G2-A2ES	1.260907631	high
TCGA-ZF-A9R4	0.664801747	low
TCGA-FD-A6TH	1.717297446	high
TCGA-E7-A7PW	0.824031893	low
TCGA-DK-A1A3	1.282550707	high
TCGA-4Z-AA80	0.90297347	low
TCGA-CF-A3MH	0.76085848	low
TCGA-XF-AAN4	1.71840288	high
TCGA-ZF-AA51	0.923278876	low
TCGA-FD-A6TK	2.186985689	high

TCGA-SY-A9G0	1.403154615	high
TCGA-XF-AAMG	1.342585947	high
TCGA-R3-A69X	1.435377202	high
TCGA-4Z-AA7N	1.028707933	low
TCGA-HQ-A5ND	1.234073144	high
TCGA-2F-A9KT	1.211546378	high
TCGA-DK-A6B2	1.168844057	high
TCGA-UY-A8OC	1.435543689	high
TCGA-ZF-AA5N	2.023552351	high
TCGA-E7-A97Q	1.186166843	high
TCGA-FD-A5BX	1.118975026	low
TCGA-GV-A3JW	1.010093682	low
TCGA-DK-A3IT	1.552260091	high
TCGA-KQ-A41N	0.813320968	low
TCGA-XF-A9SH	0.621318344	low
TCGA-C4-A0EZ	1.085153538	low
TCGA-ZF-A9R9	0.816731041	low
TCGA-C4-A0F7	1.300224885	high
TCGA-FD-A5BU	1.253247087	high
TCGA-FD-A5C1	1.587774029	high
TCGA-HQ-A2OE	0.987394208	low
TCGA-FD-A43S	0.908500851	low
TCGA-E7-A3X6	1.077680796	low
TCGA-BT-A20R	1.476824648	high
TCGA-DK-AA75	0.741206542	low
TCGA-BT-A20V	1.293019979	high
TCGA-DK-A3IV	0.835105213	low
TCGA-XF-A9T6	1.051873391	low
TCGA-4Z-AA7R	1.29735373	high
TCGA-XF-A9SU	1.154522593	high
TCGA-S5-A6DX	0.901411243	low
TCGA-2F-A9KO	0.971758065	low
TCGA-UY-A78K	0.987470398	low
TCGA-GU-A767	0.888256102	low
TCGA-DK-A1AC	1.003686455	low
TCGA-DK-A6B1	0.735034396	low
TCGA-K4-A3WS	1.300597972	high
TCGA-GU-AATQ	1.148652221	low
TCGA-YC-A89H	1.308060488	high
TCGA-ZF-AA4T	0.850329429	low
TCGA-XF-A9SJ	1.286387467	high
TCGA-FJ-A3Z9	1.057181776	low
TCGA-ZF-A9RN	0.835201084	low
TCGA-XF-A8HB	1.042396574	low
TCGA-C4-A0F0	1.534245512	high
TCGA-E5-A2PC	1.076712406	low
TCGA-DK-A6B0	0.622974808	low
TCGA-ZF-AA53	1.068354522	low
TCGA-DK-A6AW	0.680584961	low
TCGA-FD-A3SJ	1.046498333	low
TCGA-FD-A43U	1.091315216	low

TCGA-G2-A2EC	0.808964865	low
TCGA-CF-A7I0	0.895740673	low
TCGA-GC-A6I3	0.88741171	low
TCGA-BT-A42F	1.055225266	low
TCGA-XF-A8HF	0.910160088	low
TCGA-CU-A3YL	0.948160797	low
TCGA-GU-A764	1.350391247	high
TCGA-5N-A9KM	0.710808614	low
TCGA-4Z-AA81	1.285356644	high
TCGA-BL-A3JM	1.192652374	high
TCGA-FD-A6TC	1.331054277	high
TCGA-GU-A42R	1.065375571	low
TCGA-GU-A42P	1.019973764	low
TCGA-CU-A3QU	0.922547971	low
TCGA-ZF-A9RL	0.985756718	low
TCGA-FD-A3NA	1.150019342	low
TCGA-UY-A78P	1.314338807	high
TCGA-ZF-AA52	1.261053323	high
TCGA-XF-A8HC	0.670990049	low
TCGA-LT-A5Z6	1.229874814	high
TCGA-K4-A3WU	1.648319716	high
TCGA-FD-A3SQ	0.95945374	low
TCGA-ZF-AA56	1.529035854	high
TCGA-BT-A0S7	1.47609186	high
TCGA-E7-A4XJ	0.990879959	low
TCGA-E7-A519	0.470196524	low
TCGA-DK-A1AF	1.720762528	high
TCGA-ZF-A9RD	1.457284308	high
TCGA-BL-A13I	1.912367011	high
TCGA-XF-AAMY	1.198805715	high
TCGA-XF-AAMT	1.487559544	high
TCGA-UY-A9PF	0.848772807	low
TCGA-E7-A3Y1	0.585605195	low
TCGA-ZF-A9RM	1.080660458	low
TCGA-ZF-AA4V	1.419456218	high
TCGA-4Z-AA7Y	1.075795651	low
TCGA-ZF-A9R5	0.678218678	low
TCGA-DK-AA6P	1.033386416	low
TCGA-DK-A6B6	0.68882262	low
TCGA-DK-A3IK	0.875485857	low
TCGA-XF-AAN5	1.465038982	high
TCGA-E7-A6MD	1.180051688	high
TCGA-XF-A8HE	1.47585283	high
TCGA-DK-AA6M	1.154749308	high
TCGA-FD-A3SR	1.249509074	high
TCGA-CF-A3MF	0.336265942	low
TCGA-XF-A8HH	1.155080816	high
TCGA-XF-A9SI	1.247710927	high
TCGA-BT-A20Q	0.95536362	low
TCGA-E7-A97P	1.334865371	high
TCGA-YF-AA3L	0.650783378	low

TCGA-MV-A51V	1.253434484	high
TCGA-DK-A1AG	0.785501334	low
TCGA-XF-A9SZ	1.142076608	low
TCGA-CU-A5W6	1.10029801	low
TCGA-FD-A6TF	1.732843713	high
TCGA-XF-A9SP	1.292564354	high
TCGA-E7-A7DU	0.699138115	low
TCGA-FD-A6TD	1.428193145	high
TCGA-KQ-A41S	1.305415553	high
TCGA-GV-A3JV	0.943106112	low
TCGA-DK-AA6L	1.029250351	low
TCGA-G2-A3IB	1.376918835	high
TCGA-GU-A766	1.409661593	high
TCGA-XF-A9T8	1.532806807	high
TCGA-E7-A5KF	0.697106687	low
TCGA-FD-A3N5	1.736077893	high
TCGA-GU-A763	0.42916537	low
TCGA-C4-A0F1	1.187571117	high
TCGA-CF-A8HX	0.667331893	low
TCGA-ZF-A9R3	1.001295406	low
TCGA-CF-A9FM	0.542810393	low
TCGA-FD-A43N	1.044802159	low
TCGA-UY-A8OD	1.319462	high
TCGA-XF-AAMQ	1.085091949	low
TCGA-XF-A8HD	1.406919516	high
TCGA-FD-A62S	1.672423929	high
TCGA-DK-AA74	1.268721635	high
TCGA-XF-AAMZ	1.014649164	low
TCGA-XF-A9T2	1.200160187	high
TCGA-FD-A3SL	1.232916648	high
TCGA-YF-AA3M	1.10391841	low
TCGA-HQ-A2OF	0.897722388	low
TCGA-CF-A47V	0.580724624	low
TCGA-DK-A1AB	1.407448671	high
TCGA-ZF-AA58	1.268810133	high
TCGA-ZF-A9R7	0.785596388	low
TCGA-GC-A3RC	1.289565515	high
TCGA-DK-A1A5	1.431278931	high
TCGA-KQ-A41Q	0.898540314	low
TCGA-CU-A3KJ	1.22830706	high
TCGA-2F-A9KP	1.109201218	low
TCGA-FD-A62N	1.269064335	high
TCGA-ZF-A9RF	1.499272242	high
TCGA-S5-AA26	0.693092982	low
TCGA-FJ-A3Z7	0.752269994	low
TCGA-G2-A2EO	1.057578323	low
TCGA-XF-A9ST	1.2168883	high
TCGA-GC-A3RB	1.001362762	low
TCGA-DK-AA71	1.110448616	low
TCGA-FD-A3SO	1.333974357	high
TCGA-CU-A0YN	1.635266256	high

TCGA-4Z-AA7W	1.040929406	low
TCGA-ZF-A9R2	0.888849662	low
TCGA-DK-AA77	0.863008729	low
TCGA-XF-AAML	1.053855737	low
TCGA-XF-A8HI	0.870752373	low
TCGA-ZF-AA4R	1.342996911	high
TCGA-DK-A2I2	1.569560284	high
TCGA-DK-AA6R	1.669107749	high
TCGA-E7-A678	0.326846101	low
TCGA-ZF-AA4W	1.215305212	high
TCGA-PQ-A6FI	1.190304038	high
TCGA-DK-A1A6	1.126860491	low
TCGA-E7-A541	1.132401645	low
TCGA-DK-A1AE	0.899623138	low
TCGA-DK-A3X2	1.363034378	high
TCGA-DK-A3IL	1.089709987	low
TCGA-FD-A5BV	1.262568767	high
TCGA-E7-A4IJ	0.895121301	low
TCGA-E5-A4U1	0.714974779	low
TCGA-XF-AAMH	1.32623225	high
TCGA-DK-A2I6	1.295596861	high
TCGA-4Z-AA7Q	1.190151079	high
TCGA-BT-A20J	1.3082235	high
TCGA-E7-A7DV	1.613588714	high
TCGA-XF-AAMX	1.158942592	high
TCGA-GV-A3JX	1.211282475	high
TCGA-XF-AAME	1.658776888	high
TCGA-E7-A6ME	0.983996422	low
TCGA-XF-A9T0	1.00902443	low
TCGA-DK-AA6X	1.116591283	low
TCGA-CF-A47X	0.542294946	low
TCGA-FD-A6TB	1.351679212	high
TCGA-E7-A8O7	0.63807276	low
TCGA-XF-A9SV	1.244125133	high
TCGA-FJ-A3ZE	1.216315019	high
TCGA-PQ-A6FN	1.220738093	high
TCGA-FD-A3B4	1.5024125	high
TCGA-E7-A677	0.993531931	low
TCGA-UY-A78O	0.809901366	low
TCGA-FD-A43P	1.334966757	high
TCGA-XF-AAN7	1.010010995	low
TCGA-FD-A6TI	0.944458528	low
TCGA-KQ-A41P	1.028580287	low
TCGA-K4-A83P	1.157151589	high
TCGA-K4-A3WV	1.443566821	high
TCGA-BT-A3PH	1.412882468	high
TCGA-ZF-A9RC	1.134572658	low
TCGA-XF-AAMJ	1.228388125	high
TCGA-CF-A3MG	1.008845041	low
TCGA-BT-A2LB	1.064149704	low
TCGA-UY-A9PH	1.032372114	low

TCGA-DK-AA6S	1.008452508	low
TCGA-FD-A6TE	1.117549736	low
TCGA-BT-A2LA	1.173210588	high
TCGA-BT-A3PJ	1.537203693	high
TCGA-BT-A20N	1.262965435	high
TCGA-DK-A1AD	0.641748497	low
TCGA-GU-A42Q	1.435360685	high
TCGA-XF-AAN3	1.163368849	high
TCGA-UY-A9PE	1.01289166	low
TCGA-K4-A6MB	1.172349389	high
TCGA-4Z-AA7M	1.162349904	high
TCGA-K4-A54R	1.220458084	high
TCGA-XF-AAN2	1.438645016	high
TCGA-XF-A9SL	1.246090184	high
TCGA-BT-A42E	1.155582683	high
TCGA-DK-A2HX	1.534222357	high
TCGA-GU-A762	1.606668569	high
TCGA-GD-A3OS	1.544722005	high
TCGA-CF-A9FL	1.017660319	low
TCGA-FD-A3SN	1.276115006	high
TCGA-FD-A43Y	1.292723629	high
TCGA-BT-A42C	0.941048148	low
TCGA-UY-A78M	0.861914662	low
TCGA-CF-A27C	0.652189134	low
TCGA-UY-A9PD	1.002730678	low
TCGA-UY-A78L	1.426886684	high
TCGA-FD-A3B3	1.577244076	high
TCGA-DK-A3WX	1.657715923	high
TCGA-LT-A8JT	0.783143844	low
TCGA-FD-A5BS	1.194582766	high
TCGA-XF-A9T4	1.415138096	high
TCGA-G2-A2EK	0.75399603	low
TCGA-G2-A2EJ	1.656672509	high
TCGA-GC-A6I1	1.009145656	low
TCGA-G2-A3VY	0.965860713	low
TCGA-BL-A5ZZ	2.069520932	high
TCGA-4Z-AA87	0.704980333	low
TCGA-DK-A3X1	0.859209573	low
TCGA-XF-A8HG	0.652316191	low
TCGA-K4-A5RH	1.31746605	high
TCGA-DK-A1AA	0.852111748	low
TCGA-G2-AA3B	0.689196707	low
TCGA-DK-AA6Q	0.899785481	low
TCGA-DK-A1A7	0.800840817	low
TCGA-HQ-A5NE	1.544766972	high
TCGA-XF-AAN1	0.812833587	low
TCGA-FD-A62O	0.891377224	low
TCGA-4Z-AA86	1.535067834	high
TCGA-FD-A3N6	1.334395972	high
TCGA-K4-A4AB	1.160613049	high
TCGA-ZF-A9R0	1.078485679	low

TCGA-GV-A3QK	0.865651501	low
TCGA-FD-A5BR	1.085220648	low
TCGA-FJ-A3ZF	1.012100869	low
TCGA-GV-A3QI	1.236944165	high
TCGA-CF-A47W	0.600892075	low
TCGA-4Z-AA84	1.200448098	high
TCGA-BT-A20O	1.619807567	high
TCGA-XF-A9T3	2.141349248	high
TCGA-CF-A47Y	0.522615599	low
TCGA-FD-A3SP	1.673333928	high
TCGA-E5-A4TZ	1.313232593	high
TCGA-CF-A1HS	1.098009766	low
TCGA-XF-A9SK	1.014571767	low
TCGA-ZF-AA5H	2.036798781	high
TCGA-FD-A3B7	1.853499174	high
TCGA-XF-A9SX	1.2366244	high
TCGA-DK-A3IU	1.701809849	high
TCGA-C4-A0F6	1.330822295	high
TCGA-FD-A62P	1.536333555	high
TCGA-K4-A5RJ	1.358974668	high
TCGA-FD-A3B8	1.898993196	high
TCGA-ZF-AA4U	0.779378448	low
TCGA-GU-AATO	0.934933972	low
TCGA-K4-A5RI	1.16019532	high
TCGA-CF-A3MI	0.770265186	low
TCGA-CF-A9FF	0.841137209	low
TCGA-DK-AA76	0.94654927	low
TCGA-G2-A2EF	1.153923289	high
TCGA-FD-A3B6	1.410489286	high
TCGA-BT-A2LD	1.872598651	high
TCGA-GV-A40G	0.89547289	low
TCGA-XF-AAMW	1.398702151	high
TCGA-XF-A9T5	1.074068984	low
TCGA-G2-AA3C	1.146555228	low
TCGA-BT-A20T	0.976470336	low
TCGA-CU-A0YO	1.13293662	low
TCGA-H4-A2HQ	0.940447628	low
TCGA-ZF-A9RE	1.010630076	low
TCGA-GC-A3RD	1.021120753	low
TCGA-FT-A61P	1.62294934	high
TCGA-GC-A3WC	1.136465611	low
TCGA-GV-A3QF	1.028751497	low
TCGA-GD-A6C6	0.55251729	low
TCGA-UY-A9PB	1.403016453	high
TCGA-BT-A3PK	1.757160731	high
TCGA-GD-A2C5	1.160396605	high
TCGA-FT-A3EE	1.506344754	high
TCGA-CF-A8HY	0.573072662	low
TCGA-DK-A6AV	1.122334739	low
TCGA-GV-A40E	1.475490441	high
TCGA-CF-A5U8	0.417802399	low

TCGA-BT-A20W	1.238090966	high
