# Supplementary material for: Fibroblast Common Serum Response Signature-Related Classification Affects the Tumour Microenvironment and Predicts Prognosis in Bladder Cancer
Source: Oxid Med Cell Longev. 2022 Oct 19;2022:5645944. doi: 10.1155/2022/5645944 (PMC9606836; doi:10.1155/2022/5645944)
Supplement: Supplementary 7 — Supplementary Table 5: the CRS and risk stratification of GSE13507 cohort. CRS: fibroblast common serum response risk score. [file 5645944.f7.pdf]

Supplementary Table 5.The CRS and risk stratification of GSE13507 cohort.

Patient id	CRS	Risk stratification
GSM340738	2.205099931	low
GSM340668	2.207266983	low
GSM340667	2.208441284	low
GSM340695	2.211625932	low
GSM340717	2.212130182	low
GSM340670	2.213241139	low
GSM340640	2.215187117	low
GSM340643	2.215287241	low
GSM340608	2.215992793	low
GSM340740	2.217273353	low
GSM340649	2.218301893	low
GSM340625	2.220443217	low
GSM340754	2.220773024	low
GSM340658	2.220834113	low
GSM340685	2.223032051	low
GSM340718	2.224783128	low
GSM340647	2.225761492	low
GSM340726	2.226726836	low
GSM340703	2.226861714	low
GSM340621	2.226889067	low
GSM340648	2.228418158	low
GSM340724	2.229143722	low
GSM340631	2.229156836	low
GSM340719	2.230402249	low
GSM340661	2.23054099	low
GSM340730	2.231095263	low
GSM340632	2.231189219	low
GSM340635	2.231494666	low
GSM340616	2.231786277	low
GSM340626	2.233388252	low
GSM340707	2.233561208	low
GSM340657	2.233871511	low
GSM340764	2.23424663	low
GSM340659	2.235173273	low
GSM340639	2.23546046	low
GSM340747	2.235608886	low
GSM340665	2.23583512	low
GSM340617	2.236244879	low
GSM340756	2.237041181	low
GSM340686	2.237559862	low
GSM340615	2.23788553	low
GSM340748	2.23806623	low
GSM340692	2.238766643	low
GSM340650	2.239185615	low
GSM340688	2.23920078	low
GSM340630	2.239311756	low

GSM340691	2.240240241	low
GSM340749	2.240446005	low
GSM340690	2.241120425	low
GSM340624	2.241948188	low
GSM340716	2.242696462	low
GSM340644	2.242870328	low
GSM340765	2.242904804	low
GSM340762	2.24318003	low
GSM340607	2.24356647	low
GSM340750	2.243674219	low
GSM340633	2.244100258	low
GSM340739	2.244390279	low
GSM340614	2.245140788	low
GSM340743	2.245737881	low
GSM340612	2.246784847	low
GSM340721	2.247181105	low
GSM340722	2.247703043	low
GSM340713	2.247821335	low
GSM340727	2.248268357	low
GSM340627	2.248890832	low
GSM340651	2.249168011	low
GSM340736	2.250192642	low
GSM340619	2.2506084	low
GSM340613	2.250797218	low
GSM340642	2.250805173	low
GSM340757	2.251793023	low
GSM340638	2.252357829	low
GSM340737	2.253519613	low
GSM340683	2.255561637	low
GSM340611	2.255790735	low
GSM340654	2.256175088	low
GSM340699	2.257291475	low
GSM340666	2.257409412	low
GSM340620	2.257939702	low
GSM340669	2.258214517	low
GSM340741	2.258724558	low
GSM340637	2.259378869	high
GSM340623	2.259877733	high
GSM340609	2.25995648	high
GSM340753	2.260262703	high
GSM340641	2.260291538	high
GSM340735	2.26043516	high
GSM340677	2.260826218	high
GSM340693	2.260833624	high
GSM340745	2.261742822	high
GSM340682	2.262266634	high
GSM340758	2.263990956	high
GSM340768	2.264255611	high
GSM340752	2.264884544	high
GSM340689	2.265372241	high
GSM340715	2.265732107	high

GSM340634	2.266541492	high
GSM340671	2.266975616	high
GSM340653	2.267056928	high
GSM340711	2.267647025	high
GSM340710	2.267866938	high
GSM340684	2.268235228	high
GSM340723	2.268281923	high
GSM340755	2.268634563	high
GSM340678	2.269146949	high
GSM340622	2.269306021	high
GSM340698	2.269635512	high
GSM340681	2.270605243	high
GSM340655	2.271442747	high
GSM340712	2.271508829	high
GSM340733	2.271515457	high
GSM340675	2.272008785	high
GSM340751	2.272233985	high
GSM340636	2.272338586	high
GSM340652	2.272367704	high
GSM340694	2.272516943	high
GSM340679	2.273099781	high
GSM340702	2.273790872	high
GSM340662	2.274098554	high
GSM340680	2.274425586	high
GSM340700	2.275586818	high
GSM340759	2.276394984	high
GSM340676	2.276758695	high
GSM340673	2.277170298	high
GSM340610	2.277315402	high
GSM340629	2.278589708	high
GSM340697	2.278833919	high
GSM340704	2.279247461	high
GSM340664	2.279685151	high
GSM340714	2.280357424	high
GSM340645	2.282168669	high
GSM340705	2.282197916	high
GSM340656	2.282275154	high
GSM340763	2.282354293	high
GSM340708	2.282835556	high
GSM340706	2.283234864	high
GSM340672	2.284178554	high
GSM340660	2.284649568	high
GSM340767	2.287290945	high
GSM340646	2.288279906	high
GSM340734	2.28931809	high
GSM340729	2.291408187	high
GSM340663	2.292843087	high
GSM340720	2.293751758	high
GSM340760	2.293832427	high
GSM340725	2.296506952	high
GSM340744	2.296717332	high

GSM340674	2.297188184	high
GSM340605	2.298426021	high
GSM340628	2.298725706	high
GSM340701	2.30041081	high
GSM340618	2.309634917	high
GSM340687	2.31078117	high
GSM340769	2.311971288	high
GSM340742	2.317468118	high
GSM340728	2.318983432	high
GSM340761	2.323716094	high
GSM340732	2.325263325	high
GSM340766	2.332529538	high
GSM340746	2.334389772	high
GSM340606	2.336559385	high
GSM340731	2.34451315	high
GSM340696	2.345173781	high
GSM340709	2.380174227	high
