# Supplementary material for: Fibroblast Common Serum Response Signature-Related Classification Affects the Tumour Microenvironment and Predicts Prognosis in Bladder Cancer
Source: Oxid Med Cell Longev. 2022 Oct 19;2022:5645944. doi: 10.1155/2022/5645944 (PMC9606836; doi:10.1155/2022/5645944)
Supplement: Supplementary 8 — Supplementary Table 6: genes involved in cancer signalling pathways. [file 5645944.f8.doc]

Supplementary Table 6. Genes involved in cancer signalling pathways.

| Cell cycle | GINS3, MCM2, MCM3, MCM4, POLA1 |
| --- | --- |
| Negative regulation of apoptosis | AKT1, ARRB2, AVP, BCL2L1,BCL2L2, CLU, FXN, GHITM, GPX1, HGF, IGF1, LMNA, NOL3, OPA1, PARL, PPIF, PRELID1, PRKN, PSMD10, TRIAP1 |
| Positive regulation of apoptosis | BAD, BAX, BBC3, BCL2L11, BID, BIK, BMF, BNIP3, CIDEB, DNM1L, FAM162A, GPER1, HRK, MFF, MLLT11, MMP9, MOAP1, PDCD5, PINK1, PLA2G6, PLAUR, PMAIP1, PPIF, PYCARD, TNFSF10, TP53 |
| FGFR_activated | FGF1, FGF10, FGF17, FGF18, FGF19, FGF2, FGF20, FGF22, FGF23, FGF3 , FGF4, FGF5, FGF6, FGF7, FGF8, FGF9, FGFR1, FGFR2, FGFR3, FGFR4 KL, KLB |
| Hippo_activated | YAP1, TEAD1, TEAD2, TEAD3, TEAD4, WWTR1 |
| NOTCH_activated | CREBBP, EP300, HES1, HES2, HES3, HES4, HES5, HEY1, HEY2, HEYL, KAT2B, NOTCH1, NOTCH2, NOTCH3, NOTCH4, PSEN2 , LFNG, NCSTN, JAG1, APH1A, FHL1, THBS2, MFAP2, RFNG, MFAP5, JAG2, MAML3, MFNG, CNTN1, MAML1, MAML2, PSEN1, PSENEN, RBPJ, RBPJL, SNW1, ADAM10, APH1B, ADAM17, DLK1, DLL1, DLL3, DLL4, DNER, DTX1, DTX2, DTX3, DTX3L, DTX4, EGFL7 |
| PI3K_activated | EIF4EBP1, AKT1, AKT2, AKT3, AKT1S1, INPP4B,, MAPKAP1, MLST8, MTOR, PDK1, PIK3CA, PIK3CB, PIK3R2, RHEB, RICTOR, RPTOR, RPS6, RPS6KB1, STK11 |
| TGF-β_activated | TGFBR1 , TGFBR2, ACVR2A, ACVR1B, SMAD2, SMAD3, SMAD4 |
| Wnt_activated | LEF1, LGR4, LGR5, LZTR1, NDP, PORCN, SFRP1, SFRP2, SFRP4, SFRP5, SOST, TCF7L1, WIF1, ZNRF3, CTNNB1, DVL1, DVL2, DVL3, FRAT1, FRAT2, DKK1, DKK2, DKK3, DKK4, RNF43, TCF7, TCF7L2 |
| RAS_activated | ABL1, EGFR, ERBB2, ERBB3, ERBB4, PDGFRA, PDGFRB, MET, FGFR1, FGFR2, FGFR3, FGFR4, FLT3, ALK RET, ROS1, KIT, IGF1R, NTRK1, NTRK2, NTRK3, SOS1, GRB2 , PTPN11, KRAS, HRAS, NRAS, RIT1, ARAF, BRAF, RAF1, RAC1, MAP2K1, MAP2K2 ,MAPK1, INSR, INSRR, IRS1, SOS2, SHC1, SHC2, SHC3, SHC4, RASGRP1, RASGRP2, RASGRP3, RASGRP4, RAPGEF1, RAPGEF2, RASGRF1, RASGRF2, FNTA, FNTB, SPRED1, SPRED2, SPRED3, SHOC2, KSR1, KSR2, JAK2, IRS2 |
| Hippo_repressed | STK4, STK3, SAV1, LATS1, LATS2, MOB1A, MOB1B, , PTPN14, NF2, WWC1, TAOK1, TAOK2, TAOK3, CRB1, CRB2, CRB3, LLGL1, LLGL2, HMCN1, SCRIB, HIPK2, FAT1, FAT2, FAT3, FAT4, DCHS1, DCHS2, CSNK1E, CSNK1D, AJUBA, LIMD1, WTIP |
| NOTCH_repressed | ARRDC1, CNTN6, KDM5A, NOV, NRARP, ITCH, SPEN, FBXW7, HDAC2, CUL1, NCOR1, NCOR2, HDAC1, NUMB, CIR1, NUMBL, RBX1, SAP30, SKP1, CTBP1, CTBP2 |
| PI3K_repressed | DEPDC5, DEPTOR, NPRL2, NPRL3, PIK3R1, PIK3R3, PPP2R1A, PTEN, TSC1, TSC2, NF1, RASA1, CBL, ERRFI1, CBLB, CBLC , RCE1, ICMT, MRAS, PLXNB1, MAPK3, ARHGAP35, RASA2, RASA3, RASAL1, RASAL2, RASAL3, DAB2IP, PPP1CA, SCRIB , PIN1, PEBP1, ERF, PEA15 |
| Wnt_repressed | CHD8, LRP5, LRP6, RSPO1, TLE1, TLE2, TLE3, TLE4, FZD1, FZD10, FZD2, FZD3, FZD4, FZD5, FZD6, FZD7, FZD8, FZD9, WNT1, WNT10A, WNT10B, WNT11, WNT16, WNT2, WNT3A, WNT4, WNT5A, WNT5B, WNT6, WNT7A, WNT7B, WNT8A, WNT8B, WNT9A, WNT9B, AMER1, APC, AXIN1, AXIN2, GSK3B, CHD4 |
| RAS_repressed | NF1, RASA1, CBL, ERRFI1, CBLB, CBLC, RCE1, ICMT, MRAS, PLXNB1, MAPK3, ARHGAP35, RASA2, RASA3, RASAL1, RASAL2, RASAL3, DAB2IP, PPP1CA, SCRIB, PIN1, PEBP1, ERF, PEA15 |
