# Supplementary material for: Fibroblast Common Serum Response Signature-Related Classification Affects the Tumour Microenvironment and Predicts Prognosis in Bladder Cancer
Source: Oxid Med Cell Longev. 2022 Oct 19;2022:5645944. doi: 10.1155/2022/5645944 (PMC9606836; doi:10.1155/2022/5645944)
Supplement: Supplementary 9 — Supplementary Table 7: genes involved in characteristic pathway in bladder cancer. [file 5645944.f9.doc]

Supplementary Table 7. Genes involved in characteristic pathway in bladder cancer.

| Urothelial_differentiation | TMEM163, FMO9P, EPB41, IDH1, GCLC, POF1B, TBC1D1, TRAK1, BAMBI, PLCE1, SCNN1B, BHMT, DHRS2, CYB5A, PPARG, SLC9A2, TOP2B, TMEM97, NTAN1, SEMA5A, KRT20, MYCL, UPK1A, TOX3, RNF128, DAB1, ZNF436, LYPD6, ACOX1, KSR2, DMGDH, GRHL3, NIPAL1, RALGAPA2, SCNN1G, SNX |
| --- | --- |
| Ta_pathway | PLCD3, BTBD16, ANKRD20A5P, CTB-174D11.3, PROM2, SPOCD1, IGBP1, ST3GAL5, DGKA, AQP3, MAP3K5, FABP4, HNMT, MGST2, KCNQ1, IRS1, HSD17B2, ALDH1L1, SMAD3, MST1R, TCN1, CYP3A5, CTSE, HOXD1, PLCH2, SLC24A1, UGT1A1, ID3, ABCC3, PDLIM1, AGR2, NSG1, ANXA10, MR1 |
| Luminal_differentiation | FGFR3, GATA3, FOXA1, UPK2, PPARG, KRT20, SNX31, UPK1A |
| Basal_differentiation | LYNX1, SCEL, S100A8, SLPI, S100A9, PI3, CEACAM6, VSNL1, SERPINB2, CSTA, FGFBP1, SPRR1B, DSG3, KRT1, S100A7, CLCA2, LGALS7, SPRR2B, KRT6A, KRT6B, SFN, SERPINB3, KRT16, SERPINB3, SERPINB13, SERPINB4, KRT4, SPRR1A, KRT6A, ABCA12, SPRR3, TME |
| EMT_differentiation | ZEB1, ZEB2, NOTCH2, TGFB1, CDH1, SNAI2, VIM, TWIST1, SNAI1 |
| Immune_differentiation | CTLA4, CD274, PDCD1LG2, CD47, PDCD1, IDO1, MPEG1, COL17A1, CXCL11, L1CAM, SAA1 |
| Smooth_muscle | NEXN, PRUNE2, PDE5A, NEGR1, ITIH5, MSRB3, MOXD1, FGF7, FILIP1L, PTGFR, FGF7, C1S, RHOJ, TAGLN, FILIP1, ADAMTS9-AS2, LRRN4CL, NR2F1-AS1, RBPMS, PTRF, LOC100127983, PDE1A, AC131025.8, MEIS1, PELO, MYLK, ACTA2-AS1, MYH11, PDLIM7, CSGAL |
| Myofibroblasts | FCER1G, FILIP1L, FGD6, C1S, ITGB2, SAMSN1, POSTN, COL1A1, BTBD19, PTRF, MSN, SPARC, CALU, DPYSL2, CTSB, PALLD, ANXA6, STOM, MMP2, LGALS1, BGN, IFITM2, VIM, COL6A3, TGFBI, CLIC4, IFITM1, TNC, LAPTM5, CD14, LUM, AEBP1, COL3A1, DCN, NID1, SEC14L1, LAMA4, AR |
| Interferon_response | CTSB, UBE2L6, OPTN, MX1, CFB, PLSCR1, ICAM1, FCGR2A, IFIT5, CYBB, ALOX5AP, IFI44L, IFIT3, TLR2, OASL, IL15, XAF1, SP110, IFI35, NCF2, FCGR2C, CEBPB, RSAD2, IFI44, LAP3, DDX60, IFIH1, HERC6, SAMD9, HERC5, CLEC4A, PARP9, DTX3L, SAMD9L |
| Mitochondria | ALG14, SAR1B, PSMD10, TIMM10, PPA2, HNRNPC, ERH, DAD1, C1D, ATP6V0B, HINT1, DYNLL1, RAN, RPS15A, PSMB1, COX6A1, HSBP1, SERP1, EIF2S1, CAPZA2, COX7A2L, PSMA5, PSMA2, UBE2D2, VBP1, SUPT4H1, UBE2N, ZNHIT1, UQCRQ, NME1, COX7A2, COPS5, CNIH1, PSMA1, NDUFS3, NE |
| Keratinization | VSNL1, KRT14, TGM1, SERPINB4, GSDMC, KRT6A, LGALS7, SFN, SPRR2A, BG205162, C12orf54, SPRR2D, HOXD11, KRT6C, KRT5, DSG3, KRT6B, HOXD10, IL20RB, RHCG, AHNAK2, SPRR2F, FGFBP1 |
| Neuroendocrine_differentiation | ZDHHC15, FAM184A, CACNA1A, EML5, SNAP25, CACNA2D2, DCX, PSIP1, ST18, RGS7, INSM1, SLC4A8, CAMK2B, ASCL1, RTN1, SCN3A, ADAM22, FAM105A, DPY19L2P2, ASRGL1, SLC1A2, MAP6, NRXN1, TTLL7, CNKSR2, RAB39B, KCNC1, GKAP1, TMEM170B, PPM1E, ELAVL4, STXBP5L, GPR137C |
